# Comprehensive DNA methylation study identifies novel progression-related and prognostic markers for cutaneous melanoma

**DOI:** 10.1186/s12916-017-0851-3

**Published:** 2017-06-05

**Authors:** Jasper Wouters, Miguel Vizoso, Anna Martinez-Cardus, F. Javier Carmona, Olivier Govaere, Teresa Laguna, Jesuchristopher Joseph, Peter Dynoodt, Claudia Aura, Mona Foth, Roy Cloots, Karin van den Hurk, Balazs Balint, Ian G. Murphy, Enda W. McDermott, Kieran Sheahan, Karin Jirström, Bjorn Nodin, Girish Mallya-Udupi, Joost J. van den Oord, William M. Gallagher, Manel Esteller

**Affiliations:** 1 Translational Cell and Tissue Research, KU Leuven (University of Leuven), Leuven, Belgium; 2 OncoMark Ltd, NovaUCD, Dublin 4, Ireland; 3 Laboratory of Computational Biology, VIB Center for Brain & Disease Research, Leuven, Belgium; 4 Department of Human Genetics, KU Leuven (University of Leuven), Leuven, Belgium; 5 Cancer Epigenetics and Biology Program (PEBC), Bellvitge Biomedical Research Institute (IDIBELL), 08908 L’Hospitalet de Llobregat, Barcelona, Catalonia Spain; 6 Institute of Molecular Biology (IMB), Mainz, Germany; 7 Cancer Research UK, Beatson Institute, Glasgow, G61 1BD UK; 8 Department of Pathology, Maastricht University Medical Centre, Maastricht, The Netherlands; 9 Department of Surgery, St. Vincent’s University Hospital, Dublin 4, Ireland; 10 Department of Pathology and Laboratory Medicine, St. Vincent’s University Hospital, Dublin 4, Ireland; 11 Department of Clinical Sciences, Division of Pathology, Lund University, Skåne University Hospital, 221 85 Lund, Sweden; 12 UCD School of Biomolecular and Biomedical Science, UCD Conway Institute, University College Dublin, Dublin 4, Ireland; 13 Department of Physiological Sciences II, School of Medicine, University of Barcelona, Barcelona, Catalonia Spain; 14 Institucio Catalana de Recerca i Estudis Avançats (ICREA), Barcelona, Catalonia Spain

**Keywords:** Melanoma/skin cancers, Molecular diagnosis and prognosis, Molecular markers of metastasis and progression, Tumor staging, Correlation of clinical and molecular markers

## Abstract

**Background:**

Cutaneous melanoma is the deadliest skin cancer, with an increasing incidence and mortality rate. Currently, staging of patients with primary melanoma is performed using histological biomarkers such as tumor thickness and ulceration. As disruption of the epigenomic landscape is recognized as a widespread feature inherent in tumor development and progression, we aimed to identify novel biomarkers providing additional clinical information over current factors using unbiased genome-wide DNA methylation analyses.

**Methods:**

We performed a comprehensive DNA methylation analysis during all progression stages of melanoma using Infinium HumanMethylation450 BeadChips on a discovery cohort of benign nevi (n = 14) and malignant melanoma from both primary (n = 33) and metastatic (n = 28) sites, integrating the DNA methylome with gene expression data. We validated the discovered biomarkers in three independent validation cohorts by pyrosequencing and immunohistochemistry.

**Results:**

We identified and validated biomarkers for, and pathways involved in, melanoma development (e.g., *HOXA9* DNA methylation) and tumor progression (e.g., *TBC1D16* DNA methylation). In addition, we determined a prognostic signature with potential clinical applicability and validated *PON3* DNA methylation and OVOL1 protein expression as biomarkers with prognostic information independent of tumor thickness and ulceration.

**Conclusions:**

Our data underscores the importance of epigenomic regulation in triggering metastatic dissemination through the inactivation of central cancer-related pathways. Inactivation of cell-adhesion and differentiation unleashes dissemination, and subsequent activation of inflammatory and immune system programs impairs anti-tumoral defense pathways. Moreover, we identify several markers of tumor development and progression previously unrelated to melanoma, and determined a prognostic signature with potential clinical utility.

**Electronic supplementary material:**

The online version of this article (doi:10.1186/s12916-017-0851-3) contains supplementary material, which is available to authorized users.

## Background

Disruption of the epigenomic landscape is recognized as a widespread feature inherent in tumor development and progression [1, 2]. In particular, aberrant patterns of histone modifications and DNA methylation have been extensively studied because of their relevance in altering the chromatin structure and thereby also gene transcription. Specifically, research on DNA methylation changes in neoplasia has generated a multitude of biomarkers for diagnosis, prognosis, and response to treatment with application in the clinical management of several types of cancer [3].

DNA methylation changes in cancer include a wave of global DNA hypomethylation along with loci-specific hypermethylation predominantly affecting CpG islands in gene regulatory regions. Downstream transcriptional alterations have been described at all stages of tumor progression, affecting virtually all signaling pathways and unleashing a profound transformation of the cellular phenotype.

Cutaneous melanoma is the most life-threatening form of skin cancer, and its incidence and mortality keeps on rising, with the highest increase among men aged older than 55 years and women of all ages [4]. Nonetheless, clinical staging of patients with primary tumors relies entirely on classical histological biomarkers such as tumor thickness and ulceration [5]. This particular neoplasm exhibits a phenotypic plasticity that accounts for the high degree of intrinsic and acquired resistance to antineoplastic, targeted therapies, and immunotherapies [6–10]. Large-scale studies of transcriptomic alterations, along with the development of new molecular tools and in vivo models, have helped elucidate molecular cues contributing to metastasis, allowing a better understanding of melanoma biology and setting the basis for new treatment strategies [7, 11–14]. On the epigenomic side, several studies have reported DNA methylation changes in melanoma associated with inactivation of candidate tumor suppressor genes (e.g., *MAPK13*) or abnormal re-expression of oncogenes during tumor progression (e.g., TBC1D16), when examining pre-selected promoter regions for the presence of DNA methylation, or by genome-wide based approaches [15–23]. Importantly, however, the vast majority of these studies are limited to melanoma metastases and lack primary melanomas, making it problematic to identify early events during melanoma development and progression. In addition, the absence of primary tumors makes it impossible to determine DNA methylation biomarkers associated with prognosis of the patient.

Here, we present a comprehensive analysis of DNA methylation patterns during all progression stages of cutaneous melanoma. By using Infinium HumanMethylation450 BeadChips (Illumina) [24] and integrating the DNA methylome of benign nevi (n = 14) and malignant melanoma from both primary (n = 33) and metastatic (n = 28) sites with gene expression data, we identify, as well as validate in independent patient cohorts, biomarkers for melanoma development (e.g., *HOXA9* DNA methylation), tumor progression (e.g., *TBC1D16* DNA methylation), and patient prognosis (e.g., *PON3* DNA methylation and OVOL1 protein expression).

## Methods

### Patients in the discovery and validation cohorts

Fresh-frozen samples and clinical data used as the discovery cohort (n = 75) were collected at KU Leuven (Table 1). Validation cohort I, consisting of 19 primary melanomas and 23 metastases, was analyzed to validate selected biomarkers along melanoma progression. Validation cohort II, consisting of primary melanomas with clinical follow-up data provided by Lund University (Sweden), was used for the validation of the prognostic signature (Additional file 1: Table S1). A previously-constructed tissue microarray (TMA) consisting of formalin-fixed, paraffin-embedded (FFPE) primary melanomas of 179 patients with clinical follow-up data from the St. Vincent’s University Hospital (Dublin, Ireland) was used to evaluate the prognostic value of protein biomarkers (validation cohort III) [25].

**Table 1:** Characteristics of the patients included in the discovery cohort

Characteristics	No. of patients	%
All clinical samples		
Type		
Benign	14	18.6
Primary	33	44.0
Metastatic	28	37.3
Nevi		
Sex		
Male	9	64.2
Female	5	35.7
Mean age (range), years	20.6 (1–74)	
≤50	12	85.7
≥50	2	14.3
Location		
Head and neck	3	21.4
Trunk	8	57.1
Upper limbs	2	14.3
Lower limbs	1	7.1
Primary melanoma		
Sex		
Male	17	51.5
Female	16	48.5
Mean age (range), years	62.1 (34–84)	
≤50	10	30.3
≥50	23	69.7
Breslow thickness, mm		
0.01–1.0	5	15.2
1.01–2.0	8	24.2
2.01–4.0	10	30.3
>4.0	10	30.3
Clark level		
I–III	3	9.1
IV–V	30	90.9
Ulceration		
Absent	19	57.6
Present	14	42.4
Histological subtype		
Superficial spreading malignant melanoma	33	100.0
Location		
Head and neck	5	15.6
Trunk	11	34.4
Upper limb	2	6.3
Lower limb	14	43.8
Event recurrence		
Yes	14	43.8
No	18	56.3
Died of melanoma		
Yes	10	31.3
No	22	68.8
Metastatic melanoma		
Sex		
Male	9	37.5
Female	15	62.5
Mean age (range), years	60.8 (31–89)	
≤50	8	33.3
≥50	16	66.7
Breslow thickness, mm		
0.01–1.0	0	0
1.01–2.0	6	31.6
2.01–4.0	9	47.4
>4.0	4	21.1
Clark level		
I–III	2	9.5
IV–V	19	90.5
Ulceration		
Absent	8	47.1
Present	9	52.9
Histological subtype		
Superficial spreading malignant melanoma	28	100.0
Location		
Head and neck	2	10.5
Trunk	4	21.0
Upper limb	0	0
Lower limb	13	68.4

### Genome-wide DNA methylation analysis

Whole-genome DNA methylation was analyzed in the 14 normal nevi, 33 primary melanomas, and 28 melanoma metastases samples using the Illumina Infinium HumanMethylation450Beadchips. DNA was extracted from tissues by the phenol:chloroform method (only lesions with at least 75% of tumor cells were used). All DNA samples were assessed for integrity, quantity and purity by electrophoresis in a 1.3% agarose gel, PicoGreen quantification, and NanoDrop measurement. All samples were randomly distributed into 96-well plates. Bisulfite conversion of 500 ng of genomic DNA was performed using an EZ DNA methylation kit (Zymo Research) following the manufacturer’s instructions. Bisulfite converted DNA (200 ng) was used for hybridization on the HumanMethylation450 BeadChip (Illumina). Briefly, samples were whole-genome amplified followed by enzymatic end-point fragmentation, precipitation, and resuspension. The resuspended samples were hybridized onto the beadchip for 16 h at 48 °C and washed. Single nucleotide extension with labeled dideoxy-nucleotides was performed and repeated rounds of staining were carried out with a combination of labeled antibodies differentiating between biotin and dinitrophenyl. Dinitrophenyl and biotin staining, hybridization, target removal, extension, bisulfite conversion G/T mismatch, and negative and non-polymorphic control probe intensities were inspected as recommended by Illumina.

### Data analysis

#### Infinium 450 K DNA methylation data

Raw fluorescence intensity values were normalized using the minfi package in R using “preprocessIllumina” with background correction (GSE86355). Normalized intensities were then used to calculate DNA methylation levels (beta values). Likewise, data points with statistically low power (as reported by detection values of *P* > 0.01) were designated as NA and excluded from the analysis. Genotyping probes present on the chip, as well as DNA methylation probes overlapping with known single-nucleotide polymorphisms (SNPs), were also removed. Probes were considered to be in a promoter CpG island if they were located within a CpG island (UCSC database) and less than 2000 bp away from a transcription start site.

**Fig. 1:**
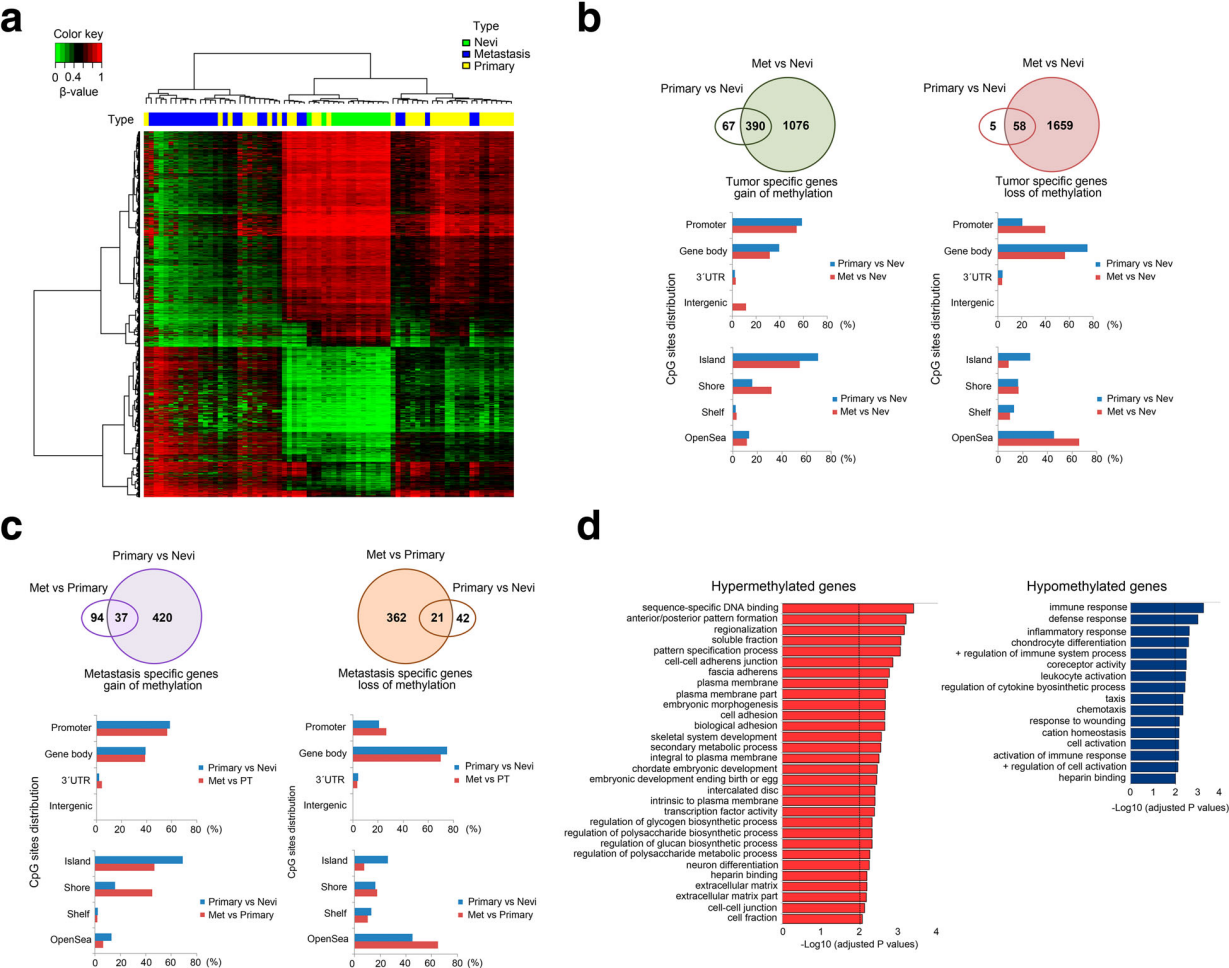
Description of DNA methylation dynamics across melanoma progression. **a** Two-dimensional clustering analysis was performed on all samples (n = 75). Probes are in rows; samples (green, nevi; yellow, primary melanomas; blue, metastases) in columns. Note that both gains and losses of DNA methylation changes occur across stages. **b** Distribution of tumor-specific DNA methylation changes in all genomic compartments: promoter, body, 3'UTR, and gene-body, and in varying CpG content and neighborhood context classified in island, shore, shelf, and open-sea. **c** Distribution of metastasis-specific DNA methylation changes in all genomic compartments: promoter, body, 3'UTR, and gene-body and in varying CpG content and neighborhood context classified in island, shore, shelf, and open-sea. **d** DAVID functional annotation of the most significant biological process categories within the hyper- (right panel) and hypomethylated (left panel) genes showing a negative correlation between DNA methylation and gene expression values (*primary* primary tumors, *meta* metastases; *P* < 0.01)

A first set of 4882 differentially methylated probes between benign nevi (n = 14), primary tumor (n = 33), and metastasis (n = 28) samples was found employing an ANOVA test. Probes were selected on the basis of showing a difference in methylation of ≥ 0.33 in at least two groups with a confidence of 0.99. Clustering in Fig. 1a was performed using the Ward method.

Epigenomic changes specific to melanomagenesis and tumor progression were detected; indeed, benign nevi, primary tumors, and metastases were separated into groups and the median of DNA methylation was computed for each probe within each group. Firstly, differences between group methylation medians (DGMB) were calculated keeping only probes with large changes (DGMB ≥ 0.25). Then, a probe-wise Mann–Whitney test was applied to further refine selected hits keeping only the statistically significant DNA methylation changes. Raw *P* values were adjusted for multiple testing using the Benjamini–Hochberg method with adjusted *P* values < 0.05 considered as significant. Hit lists from “benign nevi vs. primary melanoma” and “benign nevi vs. metastatic melanoma” comparisons were crossed to find probes that show consistent changes of DNA methylation between benign samples and tumor samples (early phase changes). Clustering of benign nevi and primary tumors (Fig. 3a left panel) was produced using the Ward method with the beta values of the DM ANOVA set (4822).

When comparing primary melanomas from patients with long (>48 months) and short survival (<48 months), the 734 differentially methylated probes were obtained by performing a non-parametric Wilcoxon–Mann–Whitney test, selecting the probes with a mean difference of ≥ 0.2 and with a corrected *P* value of < 0.01 (Fig. 3a right panel).

#### Re-analysis of public melanoma gene expression datasets

Melanoma gene expression datasets, together with raw chip data, were downloaded from the GEO database (GSE7553, GSE8401, GSE12391) [13, 26, 27]. Quality check on experiments that used Affymetrix one-channel chips were carried out with the Bioconductor package “affyQCReport”. Chips were RMA-normalized using the “affy” package and the list of differential gene expression was calculated using the package “limma”. Raw *P* values were adjusted for multiple testing according to the Benjamini–Hochberg method. Probes showing at least twofold change in gene expression with a q value smaller than 0.05 were considered significant. Dataset published by Scatolini et al. [13] used dual-color chips from Agilent combined with dye swap experiment design. Bioconductor package “limma” was used to import and normalize chips. Positive and negative control probe intensities were visualized and inspected in both channels. In addition, dye-swap chip pairs were plotted against each other and checked visually. Differential gene expression analysis was carried out using the “limma” package. Raw *P* values were adjusted according to the Benjamini–Hochberg method. Probes with at least two-fold change in gene expression and a q value smaller than 0.05 were considered significant.

### Gene ontology and gene interaction network analysis

Gene ontology analyses were performed using the web-based Database for Annotation, Visualization and Integrated Discovery (DAVID, version 6.7; david.ncifcrf.gov) [28]. Gene Set Enrichment Analysis (GSEA, version 2.04) was used to identify overrepresentation of gene sets from the online database available at the GSEA website (www.broadinstitute.org/gsea/) [29].

### Pyrosequencing

DNA methylation in clinical samples of the validation cohorts was studied by pyrosequencing, which was performed on bisulfite-treated DNA extracted from FFPE samples. Pyrosequencing reactions and quantification of DNA methylation were performed in a PyroMark Q96 System version 2.0.6 (QIAGEN) including appropriate controls. Specific primers were designed using the MethylExpress® program (Applied Biosystems) for bisulfite sequencing and PyroMark Assay Design Software (QIAGEN-version 2.0.01.15) for pyrosequencing to examine the methylation status of particular CG sites covering the promoter regions of the candidate genes (see Additional file 1: Table S2 for primer sequences).

### Immunohistochemistry (IHC)

First, primary antibodies were validated according to a previously established protocol (Additional file 2: Figures S1–S5) [30]. Briefly, antibodies obtained for each marker were checked for their specificity to the target protein by Western blotting on positive and negative control cell lines. Next, automated immunohistochemistry (IHC) using FFPE pellets of identical control cell lines was optimized to ensure specificity and to maximize differentiation between positive and negative controls (i.e., the dynamic range). Finally, IHC on whole tissue FFPE sections for the target marker and appropriate technical controls (no primary antibody and IgG from serum) were reviewed by an experienced pathologist (see Additional file 2: Figures S6, S7 for additional examples of IHC on nevi, primary melanomas, and metastases).

TMA sections were deparaffinized in xylene and rehydrated in descending gradient alcohols before heat-induced antigen retrieval in a Pre-Treatment Module (DAKO) according to the manufacturer’s instructions in citrate buffer (pH 6) or in EDTA-Tris buffer (pH 9) at 95 °C for 15 min (see Additional file 1: Table S3 for staining conditions for each primary antibody). Subsequently, immunohistochemistry was performed in a DAKO Autostainer Link 48 using an alkaline phosphatase-based EnVision G|2 System/AP Rabbit/Mouse visualization kit and Permanent Red substrate (both DAKO), resulting in a pink/red immunoreactivity. Control cell lines and conditions (see previous paragraph) were processed identically alongside the TMA.

### Automated scoring

The Aperio ScanScope XT slide scanner (Aperio Technologies) system was used to acquire whole-slide high-resolution digitized images of tissue sections with a 20× objective. Digital images were managed using Spectrum software (Aperio Technologies). The IHC-Mark image analysis software (OncoMark Ltd., Dublin, Ireland), previously validated [31, 32], was used to quantify the expression of individual markers, combining the percentage of cells stained and the intensity of the staining (H Score; see Additional file 2: Figure S8 for overview of image analysis output). Unless otherwise stated, the median H Score was used as a cutoff point to define subgroups of high or low expressing melanomas with respect to immunohistochemical markers. Melanoma-specific and progression-free survival were calculated as the interval between diagnosis of the primary tumor and melanoma-specific death or progression of the disease, respectively. Kaplan–Meier analysis and the Log-Rank statistic were generated using Graphpad Prism Version 5.02. Multivariate Cox regression analysis was performed using Statistica Version 7.

## Results

### Exploration of global methylation profiles within the discovery cohort

Genome-scale DNA methylation profiling was performed on primary (n = 33) and metastatic (n = 28) melanomas, including three paired cases, along with benign nevi (n = 14) from healthy individuals, using a previously validated DNA methylation array. The cohort consisted of melanomas with a balanced distribution among Breslow thickness, ulceration and sex, and were accompanied by detailed clinical annotation (summarized in Table 1). Importantly, to minimize intrinsic variability, only primary tumors and metastases from the most frequently occurring melanoma subtype (superficial spreading malignant melanoma; SSMM) were selected. To explore global DNA methylation profiles, clustering was performed, indicating that DNA methylation patterns clearly differentiated benign nevi from malignant melanomas into separate branches, with the exception of three primary melanomas (Fig. 1a). Two of these were thin, early-stage melanomas associated with an adjacent benign nevus (Breslow thickness < 1 mm), and the third was an *in situ* melanoma. The two other sample clusters were enriched in primary and metastatic samples, respectively, underscoring the power of DNA methylation profiles to characterize different progression stages of the disease.

### Identification of genes altered during melanoma development and progression

We next carried out a differential DNA methylation analysis to identify genes altered in melanoma development and progression. Benign nevi, primary tumors, and metastases were separated into groups and the median of DNA methylation was computed for every probe within each group. DGMB were calculated keeping only probes with large changes (DGMB ≥ 0.25), and probe-wise Mann–Whitney tests were applied to recognize statistically significant DNA methylation changes (Benjamini–Hochberg adjusted *P* < 0.05). Using these criteria, we identified 5808 probes (1533 genes) that were significantly hypermethylated in melanoma samples (primary tumors and metastases) versus benign nevi and that preferentially targeted CpG islands (primary tumors vs. nevi: 68.9% of all hypermethylated CpGs; metastases vs. nevi: 54.2%), and 4151 probes significantly hypomethylated (1722 genes) with no significant association with CpG islands (primary tumors vs. nevi: 25.8% of all hypomethylated CpGs; metastases vs. nevi: 8.4%) (two-tailed Fischer’s exact test; *P* < 0.0001) but occurring mostly in isolated CpGs in the genome (so-called ‘open sea’ CpGs; Fig. 1b and Additional file 1: Tables S4–S9 with gene lists). DNA hypermethylation affected 457 genes (77.7% of all hypermethylated genes during melanoma development and tumor progression) during melanoma development (i.e., when comparing benign nevi and primary tumors). In addition, hypermethylation prevalently affected promoter regions of genes (TSS1500, TSS200, 5UTR, 1stExon), thereby identifying 255 unique genes (55.8%) undergoing promoter hypermethylation during melanoma development (Fig. 1c, left panels). In terms of tumor progression (i.e., from primary tumors to metastases), we identified 131 differentially hypermethylated genes (22.3% of all hypermethylated genes during melanoma development and tumor progression), of which 86 (65.7%) exhibited hypermethylation at the gene promoter (Fig. 1c, left panels). There was little overlap between hypermethylated genes in primary tumors versus nevi and in metastases versus primary tumors (37 common genes), indicating that there are DNA methylation changes specific to melanoma development on the one hand and metastasis-specific DNA methylation changes linked to melanoma progression on the other. Regarding gene hypomethylation, most of the changes associated with melanoma development occurred outside gene promoters and mainly affected gene bodies, as has been previously observed in other cancer types (Fig. 1c, right panels) [33–35]. In contrast to DNA hypermethylation, loss of DNA methylation occurred at higher frequency during tumor progression (383 genes) than in melanoma development (63 genes), yet always affecting the same genomic compartments, i.e., open sea CpGs and gene bodies (Fig. 1c, right panels).

### Functional implication of DNA methylation changes in melanoma

To identify those DNA methylation changes associated with changes in gene expression, we performed an integrative analysis with gene expression profiles from benign nevi and primary and metastatic melanomas [13, 26, 27] from the GEO database (GSE7553, GSE8401, GSE12391; see Additional file 1: Tables S10–S17 for gene expression results). When comparing nevi with primary tumors and metastases, and primary tumors with metastases, we were able to examine the expression of 918 out of the 3323 unique differentially methylated genes (1536 genes hypermethylated; 1787 hypomethylated; Additional file 1: Tables S4–S9). A significant negative correlation between DNA methylation and gene expression levels was observed for 207 (22.5%) of the 918 genes at least in one of the databases analyzed. Of these, 130 genes were significantly hypermethylated and downregulated (62.8%), while 77 genes (37.2%) were similarly hypomethylated and upregulated, highlighting the importance of DNA methylation in modulating gene expression patterns (Additional file 1: Tables S18). To investigate the categories of genes exhibiting altered DNA methylation, we performed a DAVID functional annotation analysis [28]. Importantly, functional classification of the hypermethylated/downregulated genes revealed a significant involvement of several melanoma- and metastasis-related pathways, including cell/tissue polarity (GO:0009952; GO:0003002; GO:0007389) and cell-cell adhesion (GO:0005916; GO:0014704; GO:0007155; GO:0022610; GO:0005911; Fig. 1d, left panel and Additional file 1: Table S19), whereas hypomethylation-associated overexpression was enriched in GO terms involving immune system and inflammatory processes (*P* < 0.01) (GO:0006955; GO:0006952; GO:0006954; GO:0002684; GO:0045321; GO:0002253; Fig. 1d, right panel and Additional file 1: Table S20). We next used GSEA [29] to investigate which well-defined sets of genes showed significant overlap with these differentially methylated and expressed genes, and hence which sets of genes might be affected by the aberrant DNA methylation (Additional file 1: Table S21 and S22; FDR q < 0.05). Importantly, the top gene set that was found enriched in the hypermethylated/downregulated genes was JAEGER_METASTASIS_DN (30/130 genes or 23.1%), a collection of genes with downregulated expression in melanoma metastases compared to the primary tumor [36]. The next two most enriched gene sets in the hypermethylated/downregulated genes were both polycomb repressor complex 2 (PRC2) targets in human embryonic stem cells [37], corroborating previous research [38]. In addition, hypermethylated/downregulated genes typically affected genes that are downregulated in melanoma patients with a reported distant metastasis within 4 years [11] and for hypermethylated genes in lung cancer [39]. The top gene set that was found enriched in the differentially hypomethylated genes, on the other hand, was SCHUETZ_BREAST_CANCER_DUCTAL_INVASIVE_UP (13/76 genes or 17.1%), a collection of genes with upregulated expression in invasive breast cancer compared to non-invasive tumors [40]. In addition, differentially hypomethylated genes were enriched for genes that have upregulated expression in high versus low risk uveal melanomas [41].

### DNA methylation biomarkers associated with progression of melanoma

**Fig. 2:**
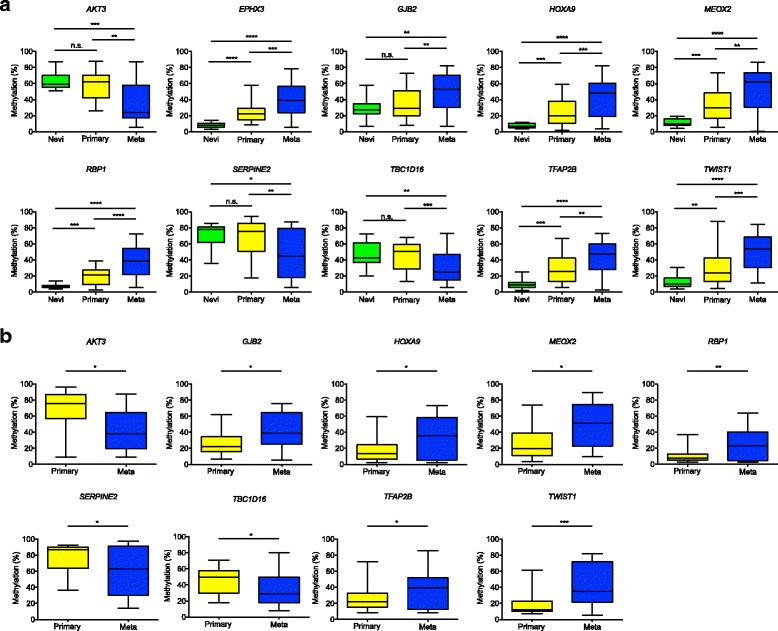
Identification of DNA methylation markers in the progression of malignant melanoma. Box-plots represent pyrosequencing results in (**a**) the discovery cohort and (**b**) the independent validation cohort I, consisting of 19 primary melanomas and 23 metastases. The selected candidates display large differences in DNA methylation between primary melanomas and metastases (DGMB ≥ 0.25), and were supported by gene expression or DNA methylation data available within publicly available databases (Additional file 1: Tables S18; *primary* primary tumors, *meta* metastases; Student’s t-test: *P < 0.05; **P < 0.01; ***P < 0.001; ****P < 0.0001).

We next searched for genes whose alteration in DNA methylation could be linked to melanoma progression in our sample cohort. Selected candidate genes exhibited (1) large differences in DNA methylation between primary melanomas and metastases (DGMB ≥ 0.25; Additional file 1: Tables S6 and S9), and (2) were supported by gene expression or DNA methylation data available within publicly available databases. Technical validation was performed aiming to compare the results provided by the original array-based epigenomic profiling and pyrosequencing. Correlation analyses showed the reliability of the screening platform used, and confirmed the suitability of pyrosequencing for validation purposes. Correlation indices between array data and pyrosequencing for the evaluated hypermethylated candidates were as follows: *EPHX3* (r = 0.81; *P* < 0.0001), *GJB2* (r = 0.71; *P* < 0.0001), *HOXA9* (r = 0.79; *P* < 0.0001), *MEOX2* (r = 0.70; *P* < 0.0001), *RBP1* (r = 0.84; *P* < 0.0001), *TFAP2B* (r = 0.68; *P* < 0.0001), and *TWIST1* (r = 0.70; *P* < 0.0001); and for the hypomethylated genes *AKT3* (r = 0.74; *P* < 0.0001), *SERPINE2* (r = 0.72; *P* < 0.0001), and *TBC1D16* (r = 0.72; *P* < 0.0001; Additional file 2: Figure S9). All of them reached statistical significance in the discovery sample set (Fig. 2a). We then conducted a validation phase by pyrosequencing of candidate epigenomically modified genes in an independent cohort of 19 primary tumors and 23 metastases (validation cohort I). DNA methylation changes linked to melanoma progression on the examined candidates retained significance in the independent validation cohort (Fig. 2b; *EPHX3* was not tested in this validation cohort).

### DNA methylation profiles identify two groups with differential melanoma-specific survival outcomes

**Fig. 3:**
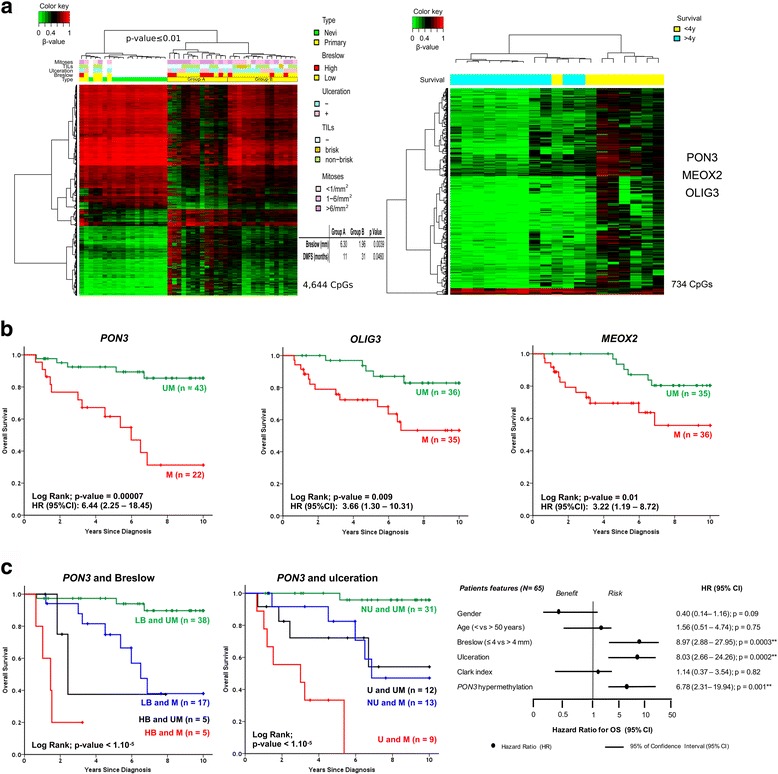
DNA methylation biomarkers with prognostic value. **a** Two groups of primary melanomas were observed in the discovery cohort when comparing primary melanomas and benign nevi, with significantly different Breslow thickness and distant metastasis-free survival (left panel); 734 probes displayed significant differences in median DNA methylation values higher than 20% when comparing the DNA methylation profiles of long survivors (>48 months) versus patients dying within this period (<48 months; right panel; *primary* primary tumor). Note that the vast majority correspond to gain-of-methylation events. **b** Kaplan–Meier survival curves for pyrosequencing results of three selected markers (*PON3*, *OLIG3*, and *MEOX2*) in validation cohort II (Additional file 1: Table S1) corroborating their prognostic power on overall survival (and progression-free survival, see Additional file 2: Figure S10; *UM* unmethylated; *M* methylated; Log-Rank test: *P* < 0.05). **c** Kaplan–Meier survival curves for *PON3* pyrosequencing results in validation cohort II grouped according Breslow thickness and ulceration status (left and middle panel, respectively; *HB* high Breslow, *LB* low Breslow, *NU* no ulceration, *U* ulceration; Log-Rank test: *P* < 0.05). Multivariate analysis for *PON3* establishes its value for survival prediction independent of these two prognostic markers (right panel; Cox regression analysis)

We next investigated whether DNA methylation could be used to predict the prognosis of patients with melanoma. We observed that the beta values of the selected 4822 probes were able to differentiate benign nevi from primary melanomas by hierarchical clustering. Among the latter, two groups of primary tumors were distinguished that clustered together according to Breslow thickness and patient survival (Fig. 3a, left panel). One group had a mean Breslow thickness of 1.96 mm and median distant metastasis-free survival of 31 months, whereas the other had significantly higher thickness and shorter survival (6.30 mm, *P* = 0.0039; 11 months, *P* = 0.0460) (no significant differences were observed for ulceration, tumor-infiltrating lymphocytes or mitotic rate; however, all primary melanomas with brisk infiltrate were clustered in group B). Given that Breslow thickness is the strongest prognostic factor in melanoma, we investigated whether the most significant, differentially methylated CpG sites could classify patients with different survival. Two DNA methylation signatures associated with 4-year survival were clearly identifiable in this respect (Fig. 3a, right panel). More than 734 probes showing significant differences in median DNA methylation values higher than 20% (*P* < 0.01) were identified when comparing the DNA methylation profiles of long survivors (>48 months) versus patients dying within this period (<48 months). The prognostic power of the markers was evaluated in an independent validation cohort containing primary melanomas (n = 85) with a balanced distribution among Breslow thickness (Additional file 1: Table S1; validation cohort II). Each of the conventional prognostic biomarkers (except age) had significant prognostic information on overall survival in this validation cohort (Additional file 1: Table S23). Differentially methylated genes included three non-melanoma related genes (*MEOX2*, *OLIG3*, *PON3*), but previously associated with DNA methylation and cancer prognosis in other pathologies [42–44]. The DNA methylation levels of the three candidates were validated by pyrosequencing in validation cohort II and survival analyses confirmed their power as indicators of overall and progression-free survival (*P* < 0.05; Fig. 3b and Additional file 2: Figure S10A, respectively). Importantly, for *PON3* DNA methylation, survival prediction was independent of the two most frequently used prognostic markers, i.e., tumor thickness according to Breslow and ulceration (*P* < 0.05; Fig. 3c and Additional file 2: Figure S10B); in addition, *PON3* DNA methylation survival prediction for progression-free survival, but not overall survival, was independent of the presence of tumor-infiltrating lymphocytes. DNA methylation of *MEOX2* and *OLIG3* did not retain significance in multivariate analysis. Moreover, DNA methylation of *PON3* was predictive for overall survival in The Cancer Genome Atlas cohort of 223 patients with melanoma [45] (Additional file 2: Figure S11). Altogether, these data constitute DNA methylation of *MEOX2*, *OLIG3*, and *PON3* as prognostic indicators potentially useful in the clinic.

### Validation of prognostic value of protein expression of differentially-methylated genes

**Fig. 4:**
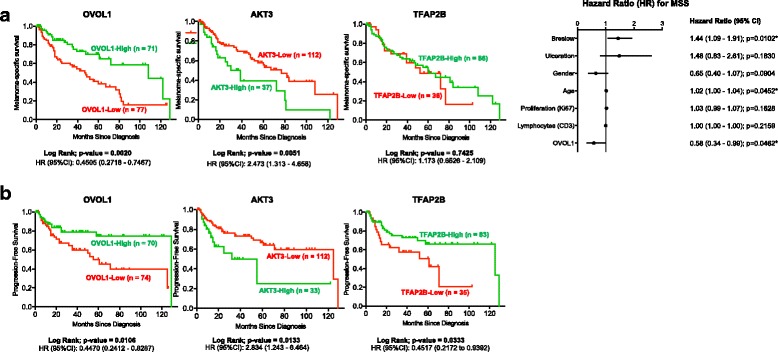
Epigenomically-regulated protein biomarkers with prognostic value. Kaplan–Meier survival curves for immunohistochemical (IHC) results of three (out of five) selected markers with differential DNA methylation (OVOL1, AKT3, and TFAP2B; results for the other two markers can be found in Additional file 2: Figure S13A, B) in the independent validation tissue microarray cohort III. The selected candidates display methylation of the promoter regions, low (or high) initial methylation levels of nevi, and a consecutive increase (or decrease) of methylation during the subsequent stages of melanoma progression. Primary antibodies were validated prior to performing IHC (Additional file 2: Figures S1–S5). Image analysis software (IHC-Mark) was used to obtain H Scores for each biomarker, combining the percentage of melanoma cells stained and the intensity of the staining. Kaplan–Meier curves together with the Log-Rank confirm the prognostic power of the protein markers on (**a**) melanoma-specific and (**b**) progression-free survival (*P* < 0.05). Multivariate Cox regression analysis manifests the value of OVOL1 protein expression in predicting melanoma-specific survival, independent of Breslow thickness (right panel in **a** and **b**). For OVOL1, the median H Score was used as a cutoff point to define subgroups of high or low expressing melanomas with respect to immunohistochemical markers; for AKT3 and TFAP2B, the third and first quartile, respectively, was used (results for AKT3 and TFAP2B with the median H Score as cutoff can be found in Additional file 2: Figure S13)

Next, we aimed to explore the possibility that the expression levels of the differentially methylated genes, linked to melanoma progression and/or prognosis, would provide prognostic information at the protein level in an independent melanoma patient cohort via IHC (validation cohort III). Candidate markers were selected applying the following criteria: (1) methylation of the promoter regions, (2) genes where initial methylation levels of nevi were low (or high), (3) consecutive increase (or decrease) of methylation during the subsequent stages of melanoma progression, and (4) availability of a high-quality antibody. Five candidate markers were selected, i.e., AKT3, EPHX3, OLIG3, OVOL1, and TFAP2B. Antibodies were validated for specificity according to a rigorous protocol [30]. In order to evaluate the prognostic value of these five markers, we performed IHC on a previously-constructed TMA consisting of archival paraffin patient samples from the St. Vincent’s University Hospital (see Additional file 2: Figure S12 for representative examples of IHC stained TMA cores with low and high expression; validation cohort III; Dublin, Ireland) [25]. Each of the conventional prognostic biomarkers had significant prognostic information on melanoma-specific survival in this TMA cohort (Additional file 1: Table S24). Image analysis software (IHC-Mark; OncoMark Ltd., Dublin, Ireland) was used to quantify the TMA stainings, combining the percentage of melanoma cells stained and the intensity of the staining (H Score). Consistent with DNA methylation data, patients with high OVOL1 expression (H Score > median H Score) in the primary tumor had significantly better prognosis than those with low expression (H Score < median H Score), displaying both extended melanoma-specific and progression-free survival (Fig. 4a, b). In addition, patients with very high AKT3 expression (H Score > third quartile H Score) in the primary tumor presented significantly worse melanoma-specific and progression-free survival than the other patients (low to moderate expression; Fig. 4a, b). Finally, patients with very low TFAP2B expression (H Score < first quartile H Score) did not have significantly different melanoma-specific survival but presented significantly shorter progression-free survival (Fig. 4a, b). EPHX3 and OLIG3 protein expression did not show any significant prognostic value in terms of survival (Additional file 2: Figure S13A, B). Importantly, multivariate Cox regression analysis validated the power of OVOL1 as an indicator of melanoma-specific survival, independent of tumor thickness according Breslow and age (*P* < 0.05; Fig. 4a, b; expression of AKT3 and TFAP2B did not retain significance in multivariate analysis). Ulceration did not retain significant prognostic value when assessed via multivariate analysis, presumably because of less standardized scoring criteria for ulceration at the time of tissue collection (from 1994 to 2007), whereas standardized scoring criteria for ulceration were only described in Europe in 2003 [46]. Altogether, these data constitute AKT3, OVOL1, and TFAP2B protein expression as prognostic indicators potentially useful in the clinic.

## Discussion

To enable the discovery of novel biomarkers and the development of more efficient therapies for melanoma, our understanding of the molecular features underlying its aggressive phenotype, and how these traits are regulated by constant modifications of its transcriptome, need to be enhanced. In this study, we aimed to profile, in an unbiased manner, DNA methylation changes occurring along the evolution of melanoma development and progression. Moreover, DNA methylation biomarkers represent a valuable tool for the clinical management of several cancer types [3]. Despite several DNA methylation changes identified in melanoma [21–23, 47], there is a lack of unbiased comprehensive analysis of clinical specimens that describes the molecular pathways targeted by epigenomic changes, and provide biomarkers that can be readily used as markers for the diagnosis and evaluation of melanoma aggressiveness. To overcome this, our study represents the most comprehensive epigenomic profiling assessment of well-annotated human melanomas. In more detail, we (1) performed genome-wide DNA methylation profiling of clinical specimens covering various stages of development and progression of SSMM; (2) integrated the observed changes with gene expression data, in order to gain insights of potential functional relevance; (3) proved the robustness of our findings through extensive validation in multiple independent cohorts; and (4) finally translated our results to potentially valuable protein biomarkers.

The present study illustrates the DNA methylation dynamics during melanoma development and progression. Aberrant DNA hypermethylation occurs predominantly in CpG island-associated promoters in melanoma cells, as compared with benign nevi. This has been described for several tumor types, and represents a common hallmark of neoplastic transformation. DNA hypomethylation, by contrast, was more frequently found at later stages of progression and predominantly associated with gene bodies, although some loci-specific changes were observed. A previous study suggested that DNA methylation alterations in melanoma could be partly attributable to the dramatic loss of 5-hydroxymethylcytosine observed during malignant progression, caused by mutation of the TET2 enzyme coding gene [48]. Altogether, a large number of DNA methylation changes were identified in relation to different stages of the disease. We were able to confirm several hypermethylated genes (see Additional file 1: Tables S4–S6 for gene lists) reported in previous studies, including transcription factor AP2 (TFAP2) genes [49], which play essential roles in the development of the epidermis and migratory cells of the neural crest, HLA-class I members [50], *SOCS-1* and -*2*, and members of the tumor necrosis factor receptor superfamily (TNFRSF) *TNFRSF10C* and *TNFRSF10D* [18], as well as *MAPK13* and *PLEKHG6* [21], and HOX family genes such as *HOXD9* [22]. We did not detect DNA methylation differences in any of the *MAGE* genes, but observed frequent hypomethylation in *TBCD1D16* [47] and in several members of the *SERPINB* gene cluster also involved in tumorigenesis (see Additional file 1: Tables S7–S9 for gene lists) [51].

By crossing our dataset with available gene expression databases, we gained insight into the potential functional relevance of DNA methylation in altering the phenotype of melanoma cells. Promoter hypermethylation of genes involved in cell adhesion, such as *ANXA9*, *CLDN5*, *GJA1*, *GJB2*, or *LAMA3*, was enriched as determined by gene ontology and GSEA analysis (Additional file 1: Tables S19 and S21), in line with previous reports (see Additional file 1: Table S18 for gene list) [52, 53]. The deregulation of cell adhesion has been recognized in other neoplasms as a characteristic event facilitating escape of the primary niche, and has been confirmed in our study by comparison with available methylation and expression databases. Loss of terminal differentiation traits, as observed by inactivation of *ESR1*, *PTPRS*, or the metastasis suppressor gene *GATA3*, may reflect the intrinsic capacity of melanoma cells to gain plasticity, and to progressively acquire changes that trigger metastatic dissemination [54, 55]. In line with this, GSEA indicated considerable and significant overlap between genes with downregulated expression in melanoma metastases compared to the primary tumor [36] and our set of differentially hypermethylated genes, and between genes with upregulated expression in invasive breast cancer compared to non-invasive tumors [40] and our differentially hypomethylated genes. The regulation of gene expression patterns by DNA methylation changes at different stages seems to reflect the phenotype switch concept that emerged from transcriptomic studies of melanomas [56–58]. Moreover, a series of studies have observed a stem-cell phenotype increasing during melanoma progression, which was strongly sustained by a tumor-promoting microenvironment [59–62]. Pathways activated by DNA hypomethylation were mostly linked to inflammation and innate or adaptive immunity processes (Additional file 1: Tables S20 and S22). Of note, although the effect of tumor-associated immune and stromal cells was minimized (by only including lesions with at least 75% of tumor cells; see Methods), some of the observed changes in DNA methylation are likely to originate from both tumor cells and normal cells. It has been hypothesized that expression of these immune and inflammatory factors in advanced melanomas interacts with the tumor microenvironment and creates a milieu supportive of tumor progression [63]. Specifically, overexpression of *TLR4* and *CCR7* in advanced melanomas as a result of loss of promoter DNA methylation fosters tumor progression by hijacking immune responses (see Additional file 1: Table S18 for gene list) [64, 65]. Further, DNA repair processes are also empowered by hypomethylation of *PARP1* (Additional file 1: Tables S8 and S18), a chromatin-associated enzyme involved in base-excision repair [66, 67]. In agreement with our data, upregulation of DNA repair pathways concomitant with a loss of cell-cell adhesion has also been reported in vertical-growth phase and metastatic melanomas in relation to regulation of NF-kappaB signaling and inhibition of apoptosis [13, 67–69].

Overall, our data support a central role for DNA methylation in modulating the transcriptome of melanoma cells, thereby changing their phenotype to promote tumor progression. At initial steps, prominent epigenomic inactivation induces loss of cell-cell contacts and truncates differentiation programs, increasing plasticity of tumor cells to acquire invasive capacities. In this line, epigenomic regulation underlies previous observations reporting downregulation of cell adhesion molecules in the most aggressive vertical-growth phase melanomas [13, 70]. Subsequently, as melanoma gains depth and invades the dermis, a transcriptional switch occurs through modulation of DNA methylation patterns leading to the epigenome displayed in the metastatic sites. DNA hypomethylation seems to be predominant at this point, and reactivation of immune and inflammation processes is evident. Upregulation of inflammation and immune response pathways in tumor cells seem to co-opt to turn the microenvironment into a tumor-promoting milieu [71, 72], and has been associated with shortened relapse-free survival [73].

Within the large panel of genes that were identified to be transcriptionally altered during melanoma progression, we selected a series of markers (*AKT3*, *EPHX3*, *GJB2*, *HOXA9*, *MEOX2*, *PON3*, *RBP1*, *SERPINE2*, *TBC1D16*, *TFAP2B*, and *TWIST1*) for further validation. The robustness of our findings was confirmed following pyrosequencing of the genes in an independent patient cohort, pointing at these alterations as widespread attributes of melanoma progression and worth further characterization. In support of this, one of the members of our gene signature, TBC1D16, has recently been shown to be involved in the metastatic cascade of melanoma [47].

A melanoma survival signature could also be inferred from this integrative study. Through a supervised correlation of the DNA methylation profiles with clinical parameters, we were able to refine a DNA methylation panel predictive of melanoma-specific survival. In line with this, significant overlap was observed, by GSEA, between our differentially hypermethylated genes and downregulated genes in melanoma patients with a reported distant metastasis within 4 years [11], and our differentially hypomethylated genes and upregulated genes in high versus low risk uveal melanomas [41]. Nowadays, prognosis for patients with clinically localized primary cutaneous melanoma relies mostly on histological parameters as tumor thickness, ulceration, and mitotic rate in the invasive component. Here, we identified, and validated in an independent validation cohort, three genes (*MEOX2*, *OLIG3*, and *PON3*) for which the degree of DNA methylation can predict the prognosis of melanoma patients. Importantly, *PON3* DNA methylation was independent of classical prognostic parameters and could, therefore, be of added value when implemented in the pathological staging procedure. In addition, we validated by IHC the prognostic usefulness of protein biomarkers (AKT3, OVOL1, and TFAP2B) that were discovered by our DNA methylation analyses, thereby verifying DNA methylomics as a valid screening tool to identify potential protein biomarkers. Furthermore, in the current era of “liquid biopsies”, the observed changes in methylation might be targets for the study of cell-free DNA in the serum of melanoma patients. Once these findings are corroborated, it could be of great utility for its clinical implementation to improve the management of melanoma patients.

## Conclusions

Our results underline the prominence of epigenomic gene regulation in eliciting metastatic spreading through the inactivation of central cancer-related pathways. Additionally, we found a panel of markers of tumor development and progression previously unrelated with melanoma, and established a prognostic signature with potential clinical utility.

## Additional files

Additional file 1: Table S1. Characteristics of patients included in validation cohort II. **Table S2.** Primers for pyrosequencing. **Table S3.** Conditions for immunohistochemical stainings. **Table S4.** Genes with differential hypermethylation in primary tumors compared to nevi. **Table S5.** Genes with differential hypermethylation in metastases compared to nevi. **Table S6.** Genes with differential hypermethylation in metastases compared to primary tumors. **Table S7.** Genes with differential hypomethylation in primary tumors compared to nevi. **Table S8.** Genes with differential hypomethylation in metastases compared to nevi. **Table S9.** Genes with differential hypomethylation in metastases compared to primary tumors. **Table S10.** Differential gene expression results of the comparison between nevi and radial growth phase primary melanoma (GSE12391, 0.05 and fold change > 2). Positive logFC means higher expression in radial growth phase primary melanoma. **Table S11.** Differential gene expression results of the comparison between nevi and vertical growth phase primary melanoma (GSE12391, 0.05 and fold change > 2). Positive logFC means higher expression in vertical growth phase primary melanoma. **Table S12.** Differential gene expression results of the comparison between nevi and metastases (GSE12391, 0.05 and fold change > 2). Positive logFC means higher expression in nevi. **Table S13.** Differential gene expression results of the comparison between metastases and radial growth phase primary melanoma (GSE12391, 0.05 and fold change > 2). Positive logFC means higher expression in metastases. **Table S14.** Differential gene expression results of the comparison between metastases and vertical growth phase primary melanoma (GSE12391, 0.05 and fold change > 2). Positive logFC means higher expression in metastases. **Table S15.** Differential gene expression results of the comparison between primary melanomas and metastases (GSE7753, 0.05 and fold change > 2). Positive logFC means higher expression in metastases. **Table S16.** Differential gene expression results of the comparison between primary tumors and metastases (GSE8401, 0.05 and fold change > 2). Positive logFC means higher expression in metastases. **Table S17.** Gene lists of differentially methylated and expressed genes. **Table S18.** Gene list of hypermethylated/downregulated and hypomethylated/upregulated genes. **Table S19.** DAVID functional annotation analysis of differentially hypermethylated and expressed genes. **Table S20.** DAVID functional annotation analysis of differentially hypomethylated and expressed genes. **Table S21.** Gene Set Enrichment Analysis of differentially hypermethylated and expressed genes. **Table S22.** Gene Set Enrichment Analysis of differentially hypomethylated and expressed genes. **Table S23.** Results for univariate analyses of conventional prognostic biomarkers in validation cohort II. **Table S24.** Results for univariate analyses of conventional prognostic biomarkers in validation cohort III. (XLS 10860 kb)

Additional file 2: Figures S1–S5. Validation of primary antibodies against AKT3, EPHX3, OLIG3, OVOL1, and TFAP2B, respectively, according a previously established protocol (Gillian O’Hurley, Molecular Oncology, 2014). First, antibodies obtained for each marker were checked for their specificity to the target protein by western blot on positive and negative control cell lines. Next, automated immunohistochemistry (IHC) using formalin-fixed, paraffin-embedded (FFPE) pellets of identical control cell lines was optimized to ensure specificity and to maximize differentiation between positive and negative controls (i.e., the dynamic range). Finally, IHC on whole tissue FFPE sections for the target marker and appropriate technical controls (no primary antibody and IgG from serum) were reviewed by an experienced pathologist. **Figure S6, S7.** Representative examples of IHC on nevi, primary melanomas and metastases. **Figure S8.** (A) Examples of original tissue microarray (TMA) core and mark-up image for varying, indicated H Scores as output from IHC-Mark image analysis software. (B) Overview graphs indicating the density plots of IHC-Mark image analysis H Score for each protein marker. **Figure S9.** Correlation plots and indices of the technical validation comparing the original array-based epigenomic profiling and pyrosequencing. **Figure S10.** (A) Kaplan–Meier survival curves for pyrosequencing results of three selected markers (*PON3*, *OLIG3*, and *MEOX2*) in validation cohort II (Additional file 1: Table S1) corroborating their prognostic power on progression-free survival (and overall survival, see Fig. 3; UM, unmethylated; M, methylated; Log-Rank test: *P* < 0.05). (B) Kaplan–Meier survival curves for *PON3* pyrosequencing results grouped according Breslow thickness and ulceration status (left and middle panel, respectively; HB, high Breslow; LB, low Breslow; NU, no ulceration; U, ulceration; No, no tumor-infiltrating lymphocytes (TILs) present; TILs, TILs present; Log-Rank test: *P* < 0.05). Multivariate analysis for *PON3* establishes its value for survival prediction independent of these two prognostic markers (right panel; Cox regression analysis). **Figure S11.** Kaplan–Meier survival curve for the DNA methylation of *PON3* as a predictor for 2-year overall survival in The Cancer Genome Atlas cohort of 223 patients with melanoma (UM, unmethylated; M, methylated; Log-Rank test: *P* < 0.05). **Figure S12.** Representative examples of immunohistochemically stained TMA cores with low and high expression for each biomarker. **Figure S13.** Kaplan–Meier survival curves for IHC results of four (out of five) selected markers with differential DNA methylation (AKT3, EPHX3, OLIG3, and TFAP2B; results for the other two markers can be found in Fig. 4a, b) in the independent validation tissue microarray cohort III. The selected candidates display methylation of the promoter regions, low (or high) initial methylation levels of nevi, a consecutive increase (or decrease) of methylation during the subsequent stages of melanoma progression. Primary antibodies were validated prior to performing IHC (Additional file 2: Figures S1–S5). Image analysis software (IHC-Mark) was used to obtain H Scores for each biomarker, combining the percentage of melanoma cells stained and the intensity of the staining. Kaplan–Meier curves display the analysis of their prognostic power on (A) melanoma-specific and (B) progression-free survival (*P* < 0.05). For all markers, the median H Score was used as a cutoff point to define subgroups high or low expressing melanomas. (PDF 67139 kb)

## Abbreviations

DAVID: Database for Annotation, Visualization and Integrated Discovery
DGMB: differences between group methylation medians
FFPE: formalin-fixed, paraffin-embedded
GO: gene ontology
GSEA: gene set enrichment analysis
IHC: immunohistochemistry
SNP: single-nucleotide polymorphism
SSMM: superficial spreading malignant melanoma
TMA: tissue microarray
