# Supplementary material for: Comprehensive DNA methylation study identifies novel progression-related and prognostic markers for cutaneous melanoma
Source: BMC Med. 2017 Jun 5;15:101. doi: 10.1186/s12916-017-0851-3 (PMC5458482; doi:10.1186/s12916-017-0851-3)
Supplement: Supplementary file 2 — Validation of primary antibodies against AKT3, EPHX3, OLIG3, OVOL1, and TFAP2B, respectively, according a previously established protocol (Gillian O’Hurley, Molecular Oncology, 2014). First, antibodies obtained for each marker were checked for their specificity to the target protein by western blot on positive and negative control cell lines. Next, automated immunohistochemistry (IHC) using formalin-fixed, paraffin-embedded (FFPE) pellets of identical control cell lines was optimized to ensure specificity and to maximize differentiation between positive and negative controls (i.e., the dynamic range). Finally, IHC on whole tissue FFPE sections for the target marker and appropriate technical controls (no primary antibody and IgG from serum) were reviewed by an experienced pathologist. Figure S6, S7. Representative examples of IHC on nevi, primary melanomas and metastases. Figure S8. (A) Examples of original tissue microarray (TMA) core and mark-up image for varying, indicated H Scores as output from IHC-Mark image analysis software. (B) Overview graphs indicating the density plots of IHC-Mark image analysis H Score for each protein marker. Figure S9. Correlation plots and indices of the technical validation comparing the original array-based epigenomic profiling and pyrosequencing. Figure S10. (A) Kaplan–Meier survival curves for pyrosequencing results of three selected markers (PON3, OLIG3, and MEOX2) in validation cohort II (Additional file 1: Table S1) corroborating their prognostic power on progression-free survival (and overall survival, see Fig. 3; UM, unmethylated; M, methylated; Log-Rank test: P < 0.05). (B) Kaplan–Meier survival curves for PON3 pyrosequencing results grouped according Breslow thickness and ulceration status (left and middle panel, respectively; HB, high Breslow; LB, low Breslow; NU, no ulceration; U, ulceration; No, no tumor-infiltrating lymphocytes (TILs) present; TILs, TILs present; Log-Rank test: P < 0.05). Multivariate analysis for PON3 [file 12916_2017_851_MOESM2_ESM.pdf]

Supplementary Figure 1

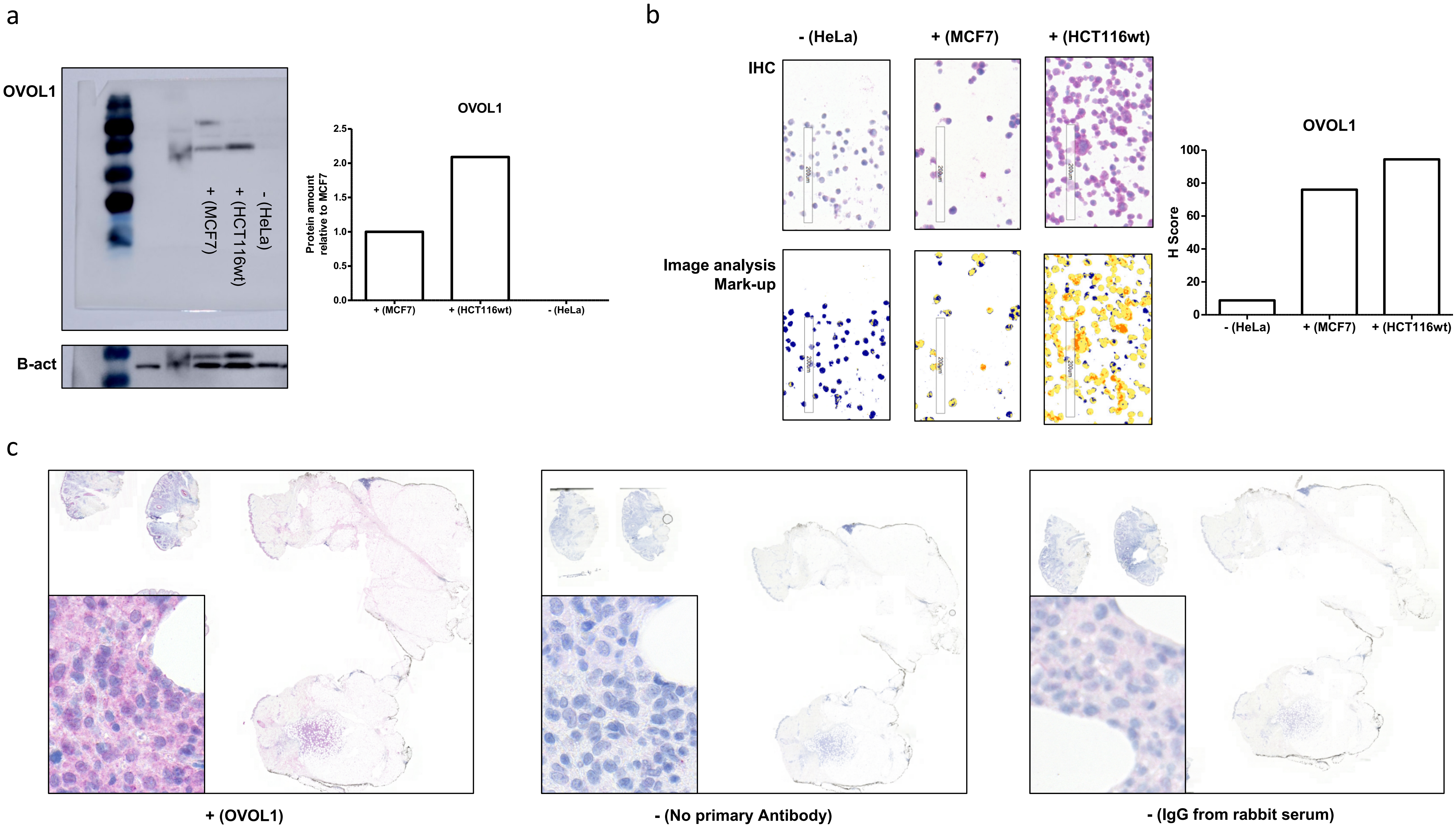

Supplementary Figure 2

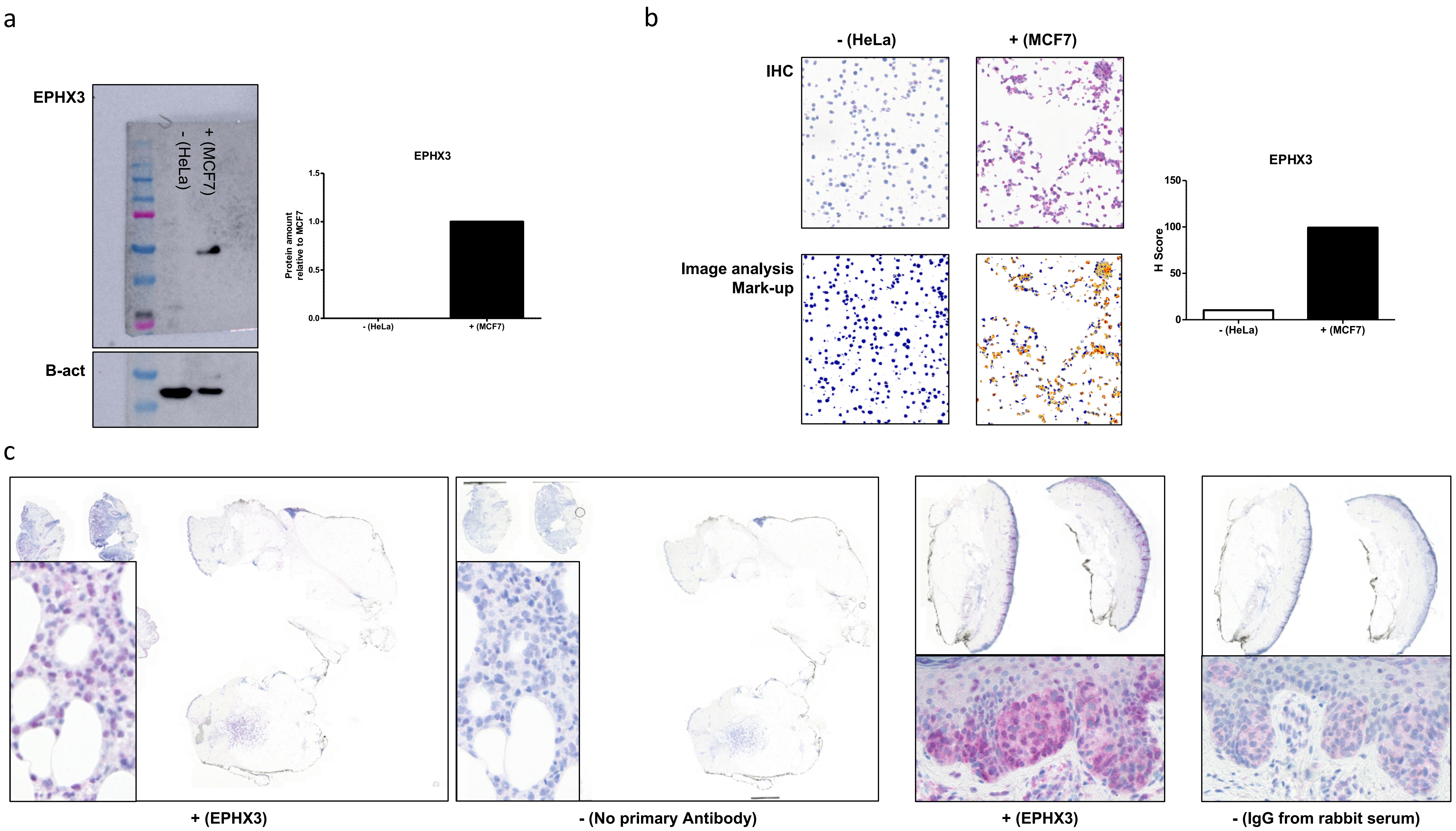

Supplementary Figure 3

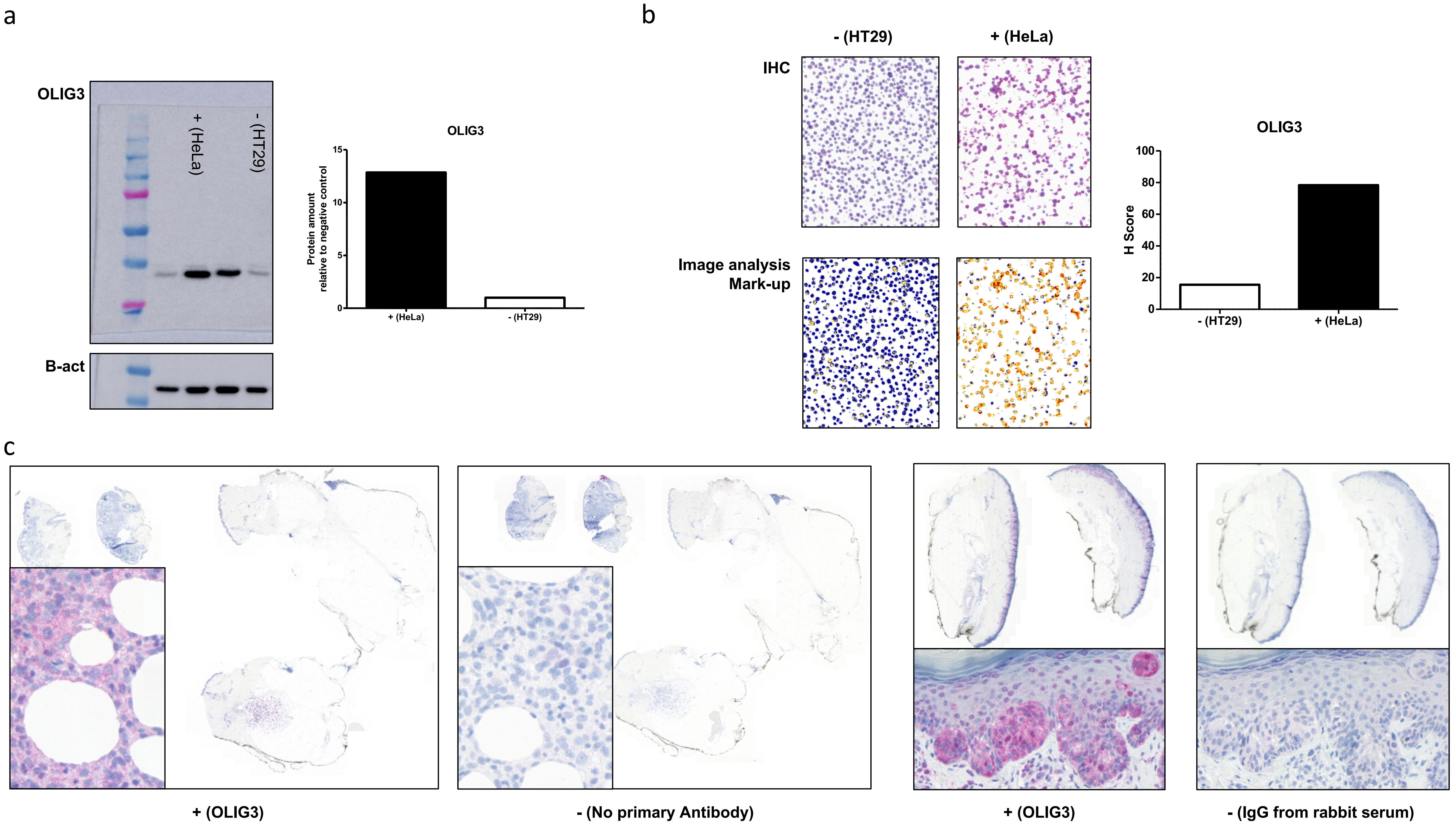

Supplementary Figure 4

a

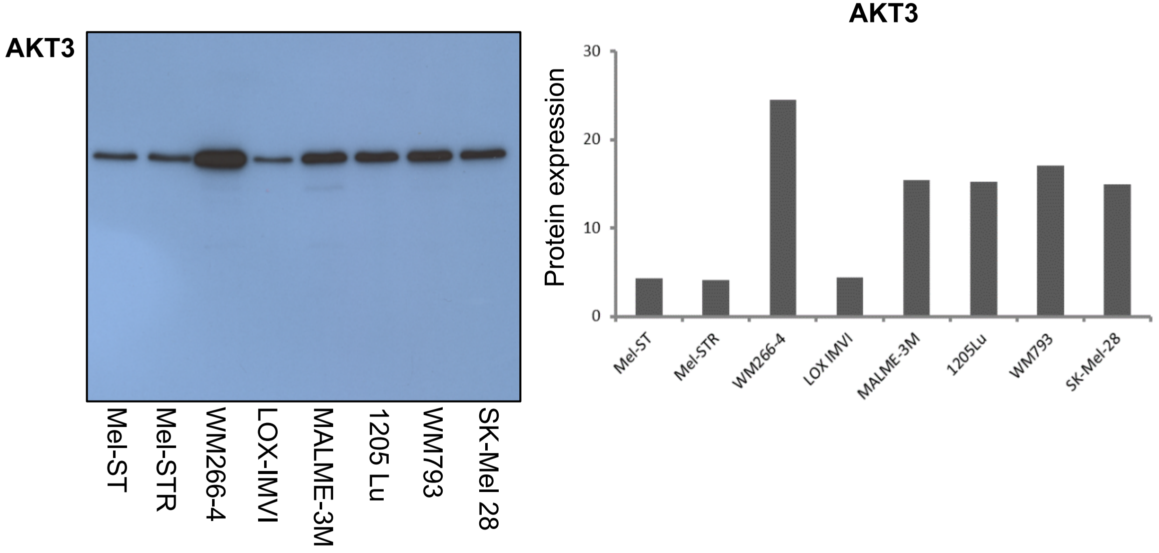

b

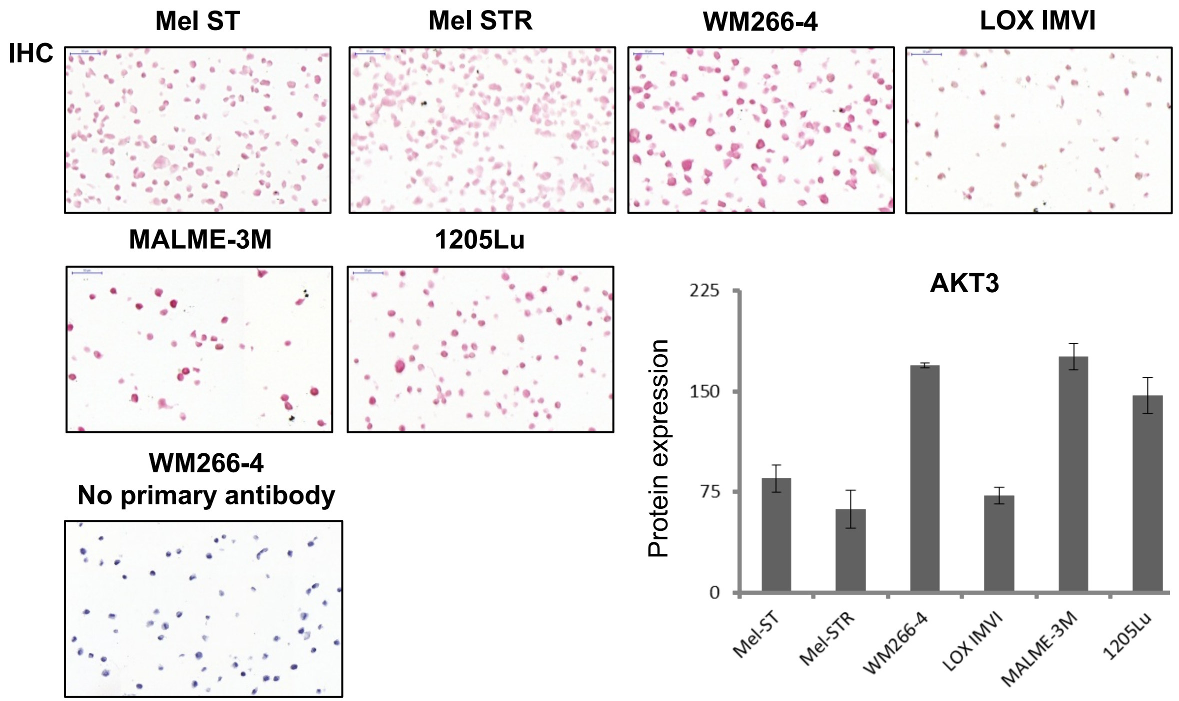

c

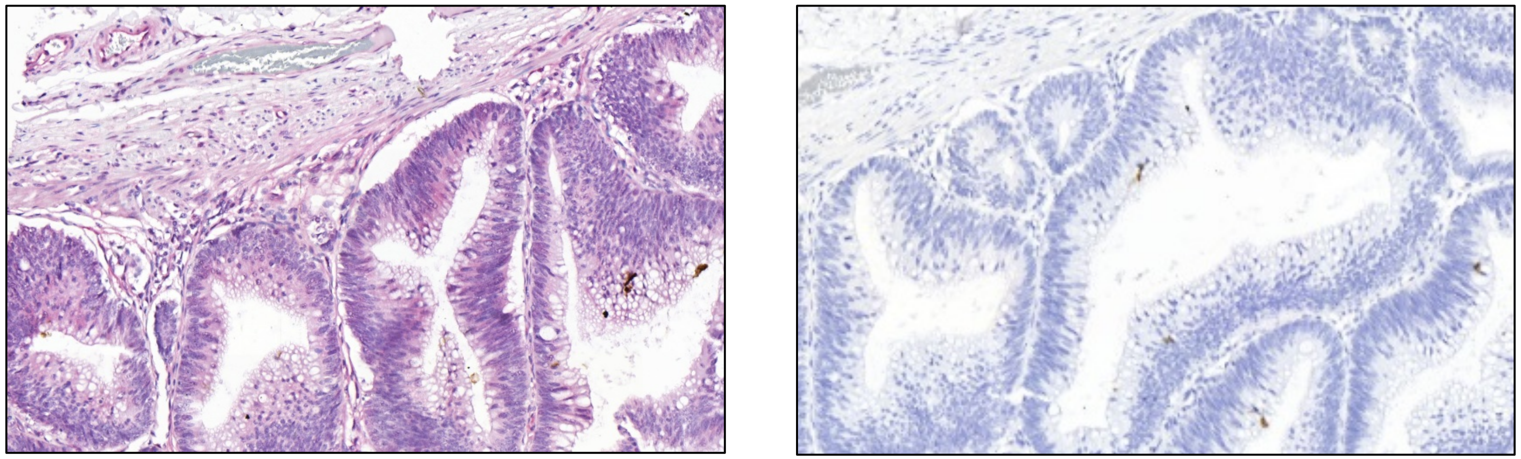

+ (AKT3)

- (No primary Antibody)

Supplementary Figure 5

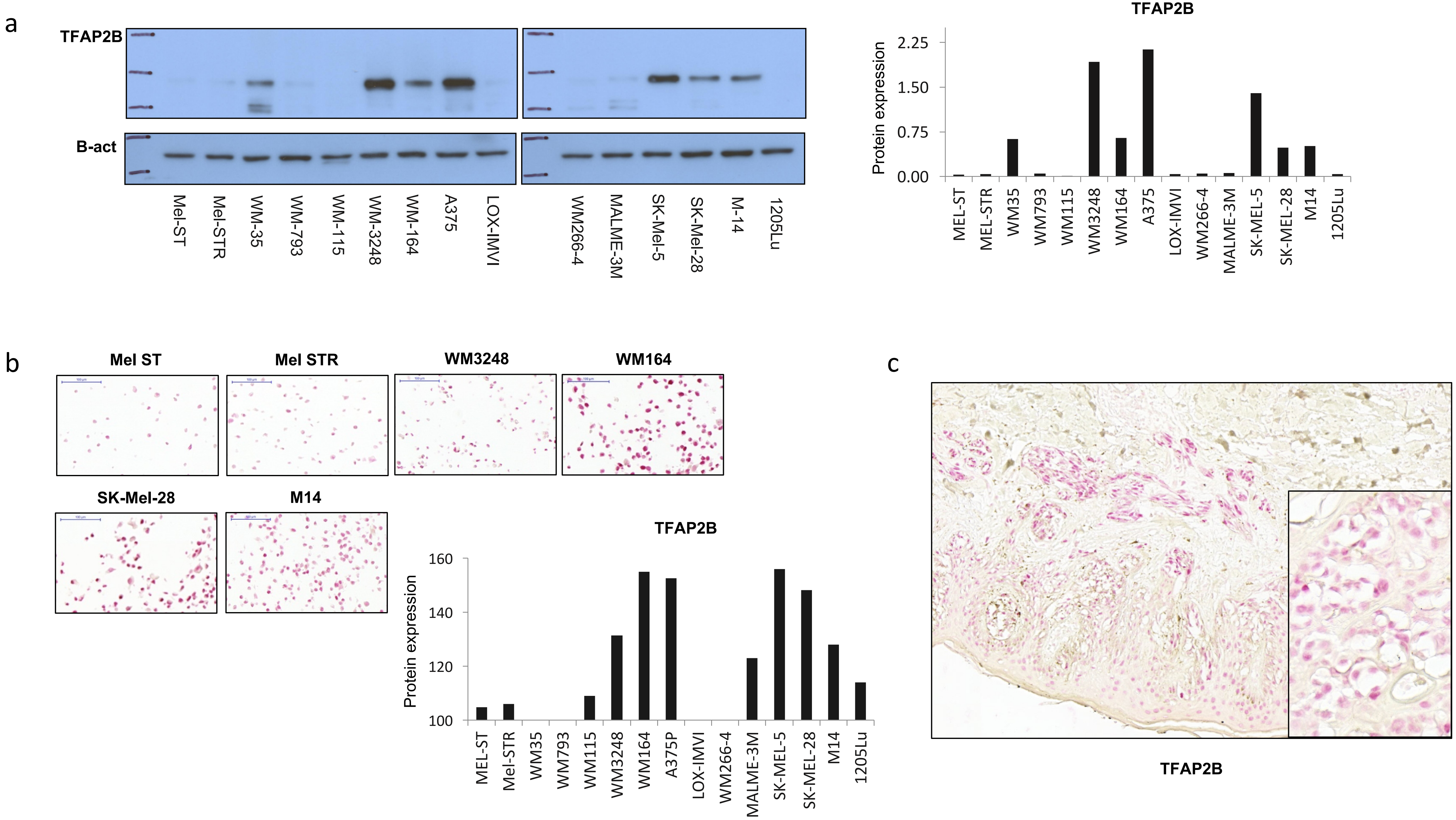

Supplementary Figure 6

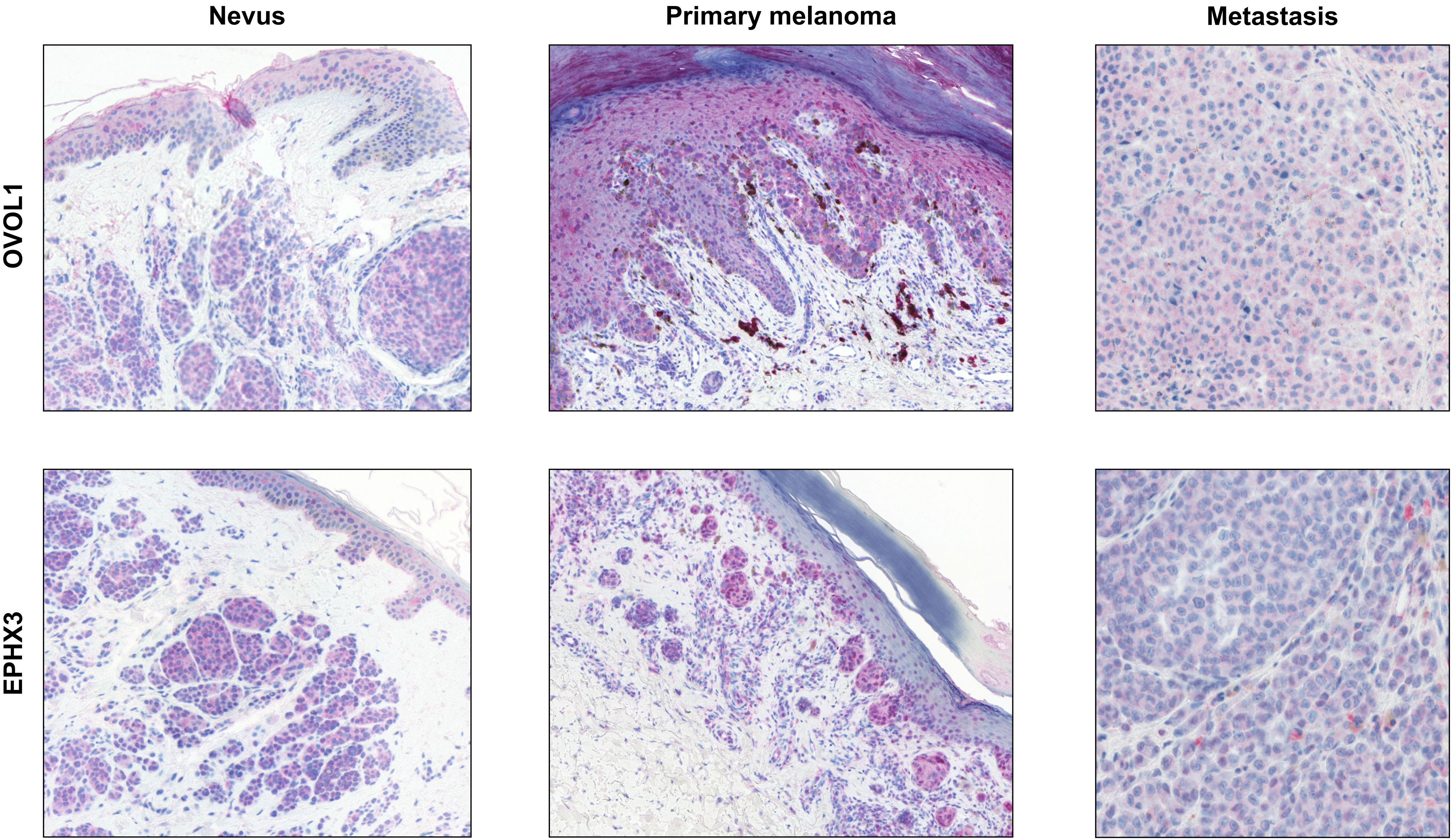

Supplementary Figure 7

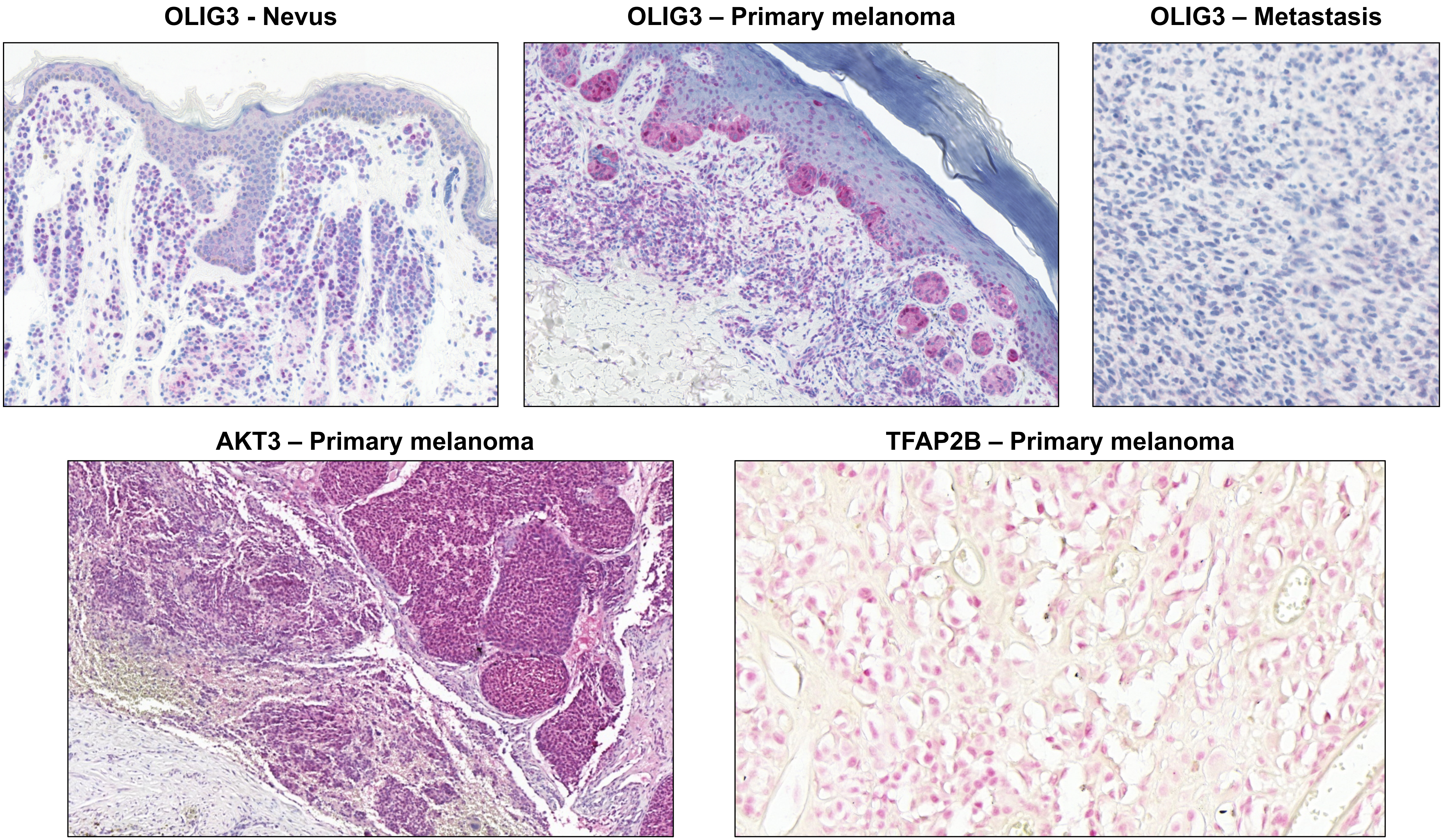

Supplementary Figure 8

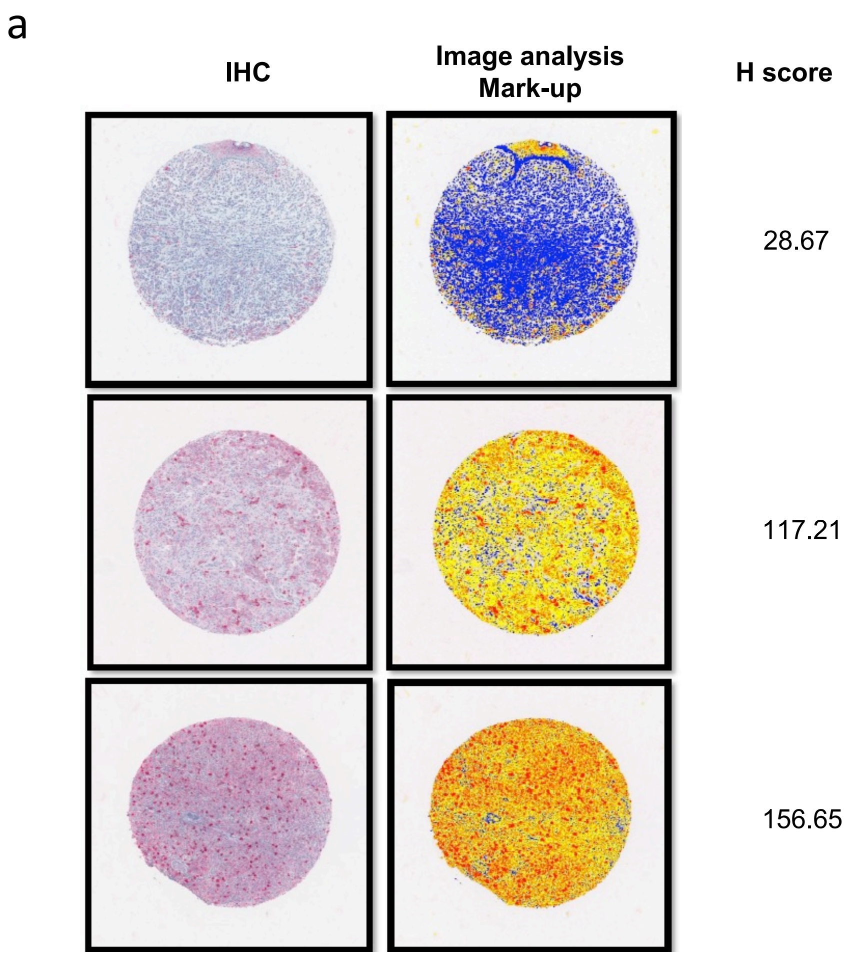

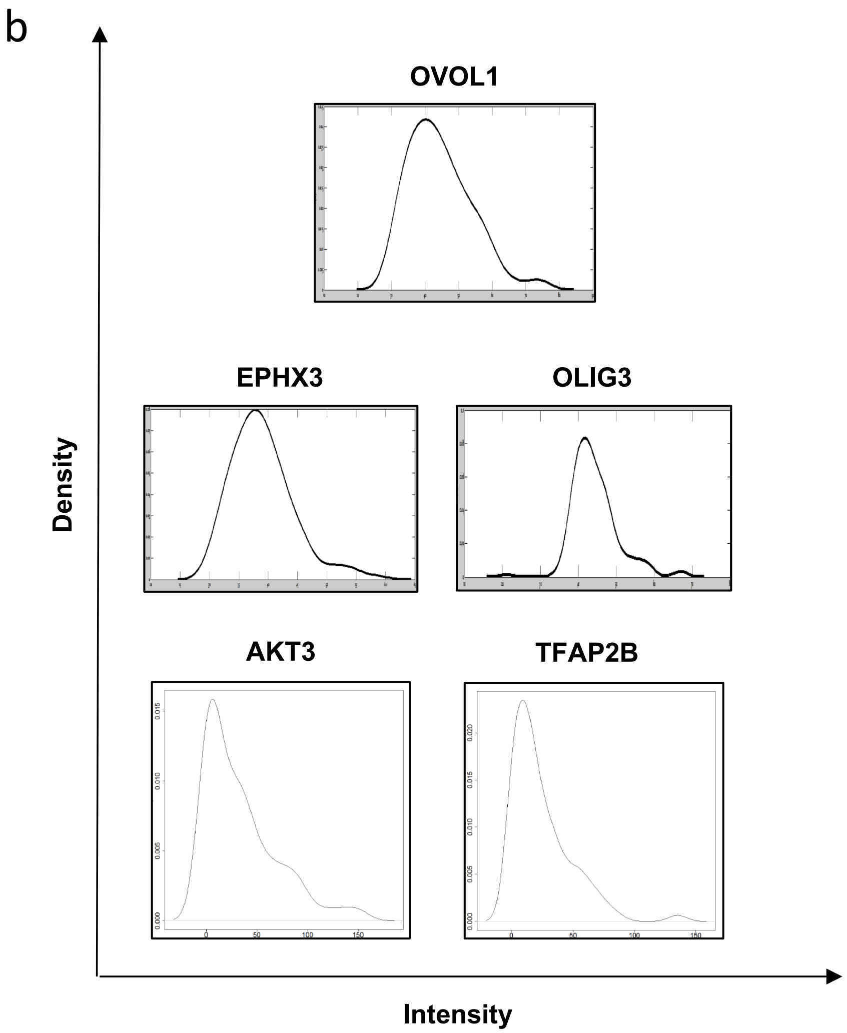

Supplementary Figure 9

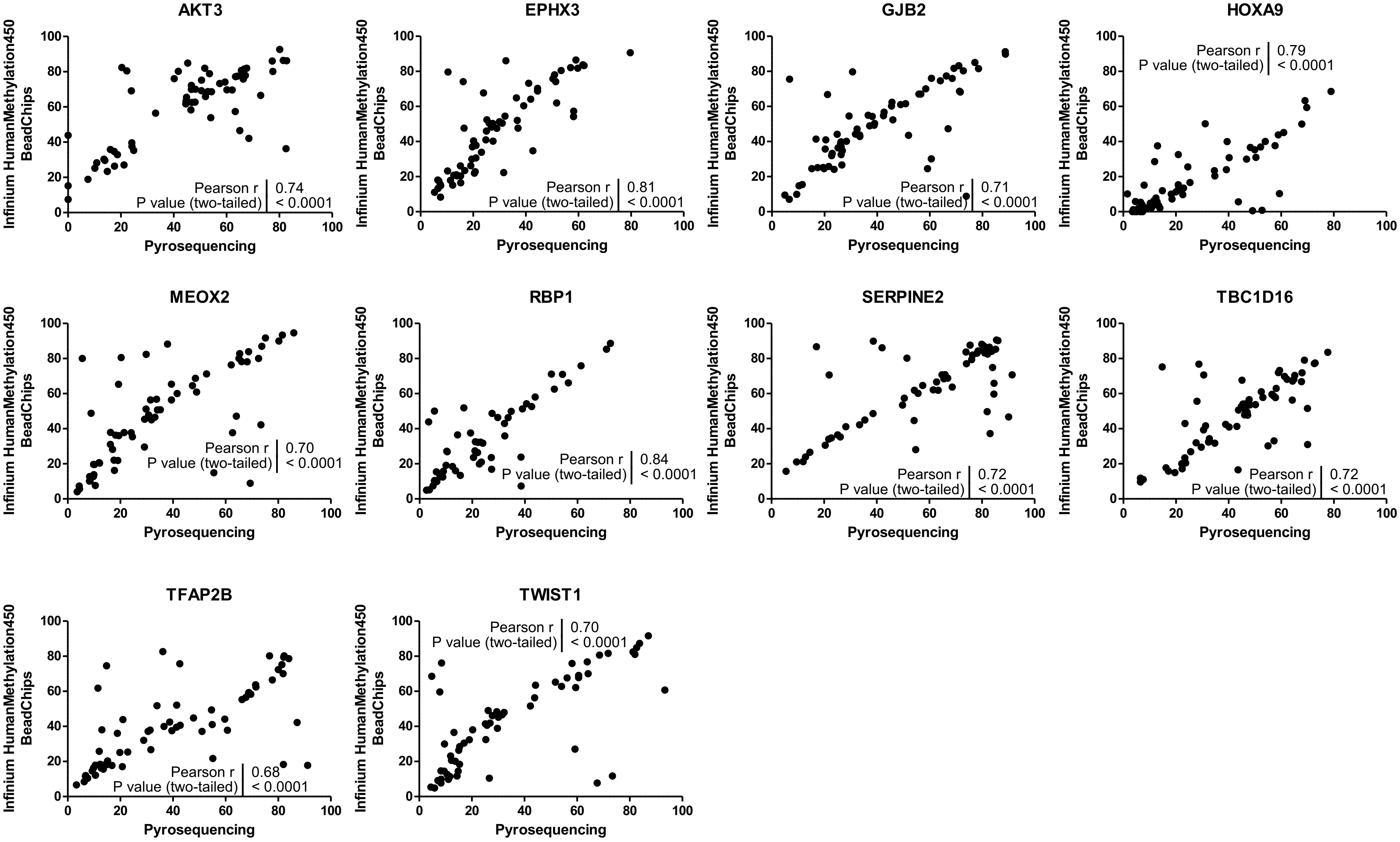

Supplementary Figure 10

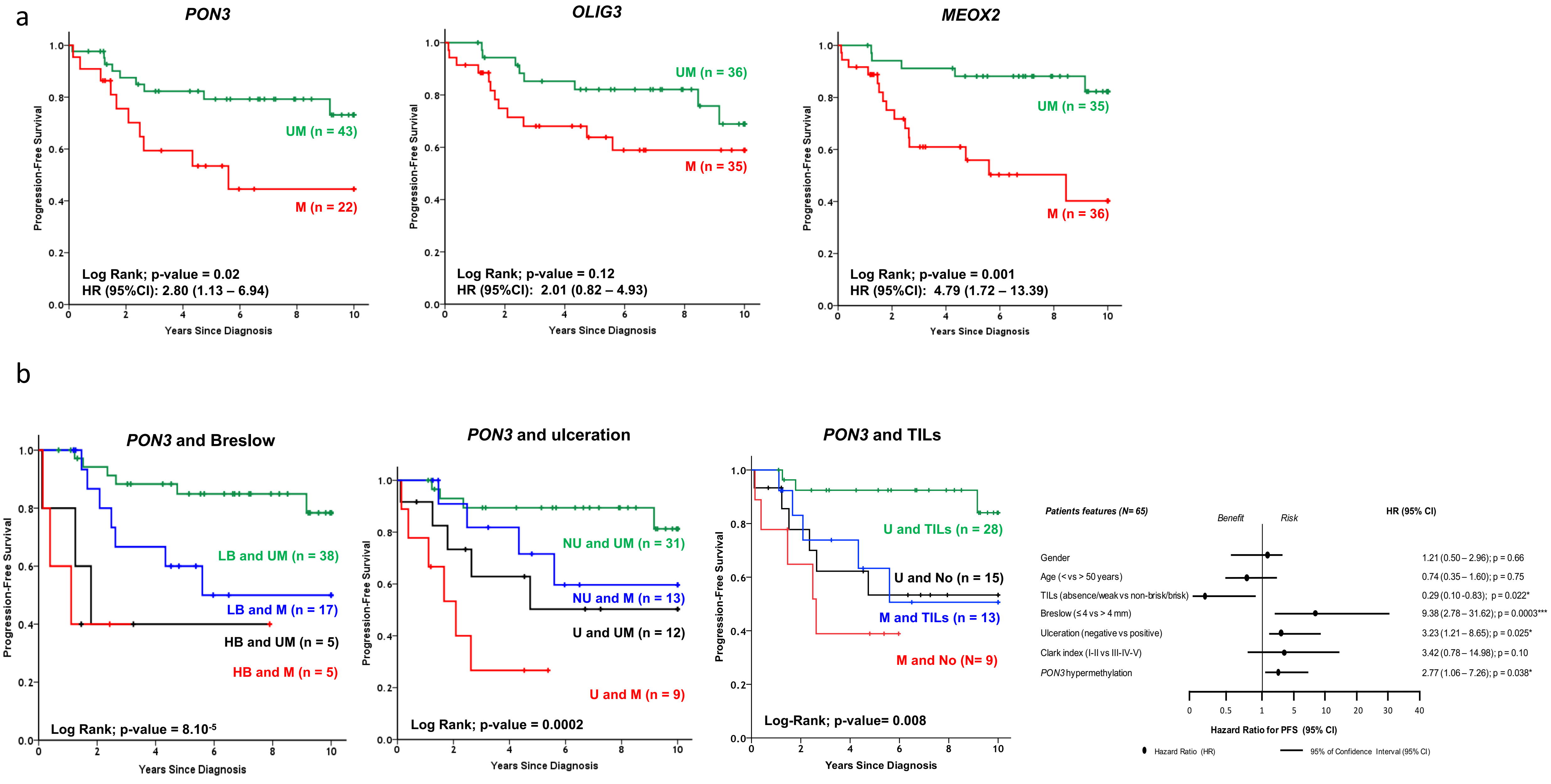

Supplementary Figure 11

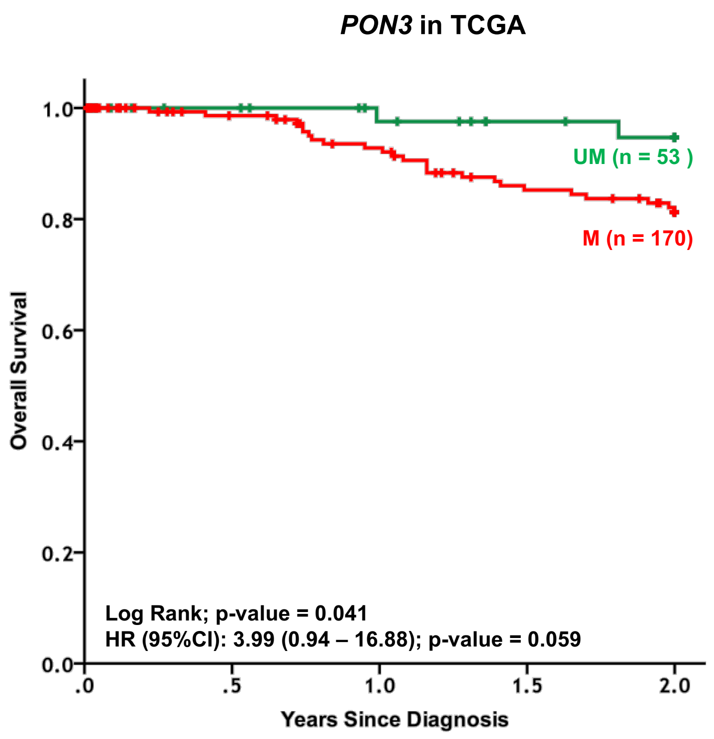

Supplementary Figure 12

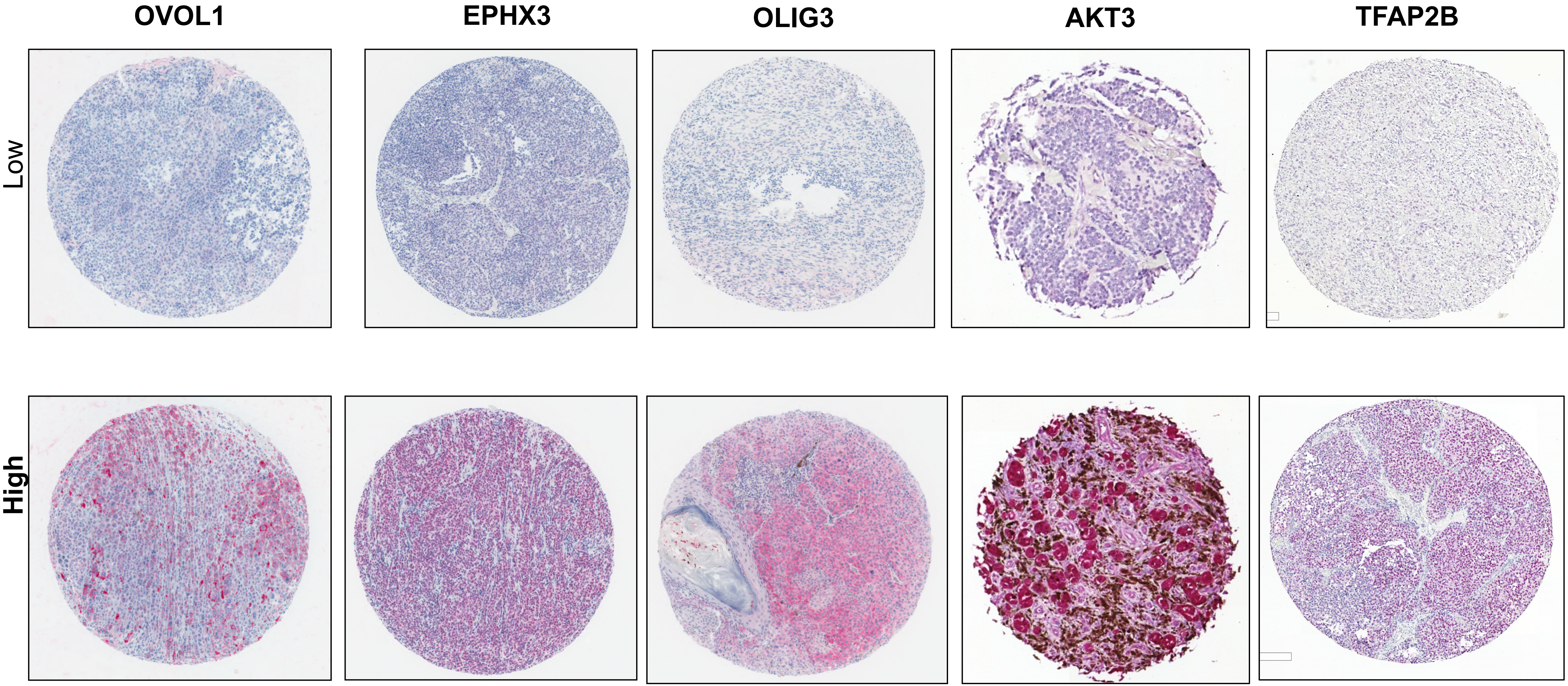

Supplementary Figure 13

a

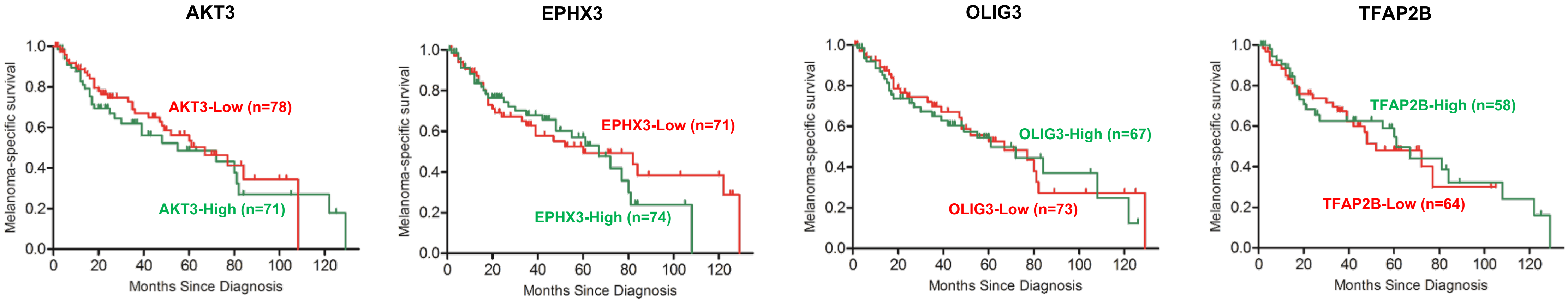

Log Rank; p-value = 0.4920
HR (95%CI): 1.197 (0.7165 – 2.000)

Log Rank; p-value = 0.7142
HR (95%CI): 1.103 (0.6540 – 1.859)

Log Rank; p-value = 0.8049
HR (95%CI): 1.069 (0.6307 – 1.811)

Log Rank; p-value = 0.7696
HR (95%CI): 0.9191 (0.5226 – 1.616)

b

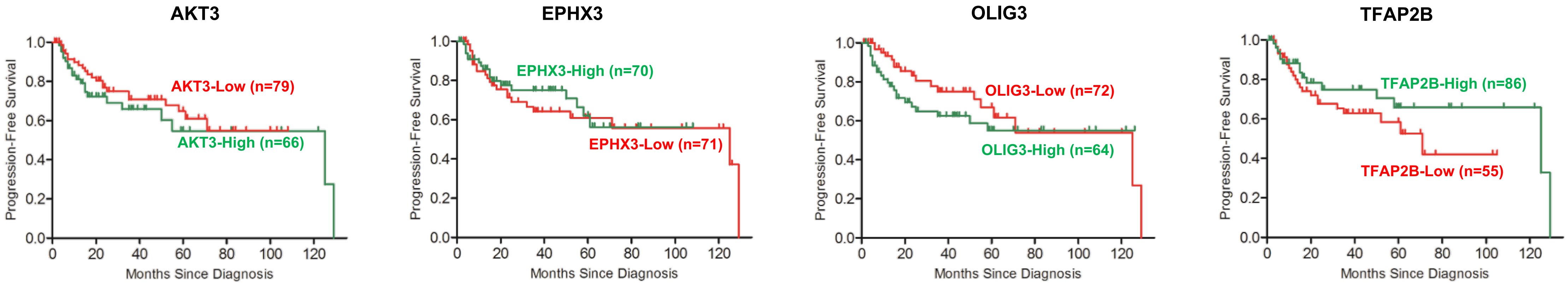

Log Rank; p-value = 0.3964
HR (95%CI): 1.315 (0.6986 – 2.474)

Log Rank; p-value = 0.7432
HR (95%CI): 0.8995 (0.4774 – 1.695)

Log Rank; p-value = 0.2063
HR (95%CI): 1.504 (0.7986 – 2.833)

Log Rank; p-value = 0.2302
HR (95%CI): 0.6642 (0.3404 – 1.296)
